# Differential regulatory control of curli (*csg*) gene expression in *Salmonella enterica* serovar Typhi requires more than a functional CsgD regulator

**DOI:** 10.1038/s41598-023-42027-y

**Published:** 2023-09-09

**Authors:** Camille Ou, Charles M. Dozois, France Daigle

**Affiliations:** 1 Department of Microbiology, Infectiology and Immunology, University of Montréal, 2900 Bd Édouard-Montpetit, Montreal, QC H3T 1J4 Canada; 2 CRIPA, Centre de Recherche en Infectiologie Porcine et Avicole, Faculté de Médecine Vétérinaire, 3200 Sicotte, St-Hyacinthe, QC J2S 2M2 Canada; 3 Centre Armand-Frappier Santé Biotechnologie, Institut Nationale de la Recherche Scientifique (INRS), 531 Boul des Prairies, Laval, QC H7V 1B7 Canada

**Keywords:** Bacterial pathogenesis, Bacteriology

## Abstract

The human-specific *Salmonella enterica* serovar Typhi (*S.* Typhi) causes typhoid fever, a systemic disease with no known reservoir. Curli fimbriae are major components of biofilm produced by *Salmonella* and are encoded by the *csg* gene cluster (*csgBAC* and *csgDEFG*). The role of curli in *S.* Typhi is unknown, although detection of anti-curli antibodies suggests they are produced during host infection. In this study, we investigated curli gene expression in *S.* Typhi. We demonstrated that the CsgD regulatory protein binds weakly to the *csgB* promoter. Yet, replacing *S.* Typhi *csgD* with the *csgD* allele from *S.* Typhimurium did not modify the curli negative phenotype on Congo Red medium suggesting that differential regulation of curli gene expression in *S.* Typhi is not dependent on modification of the CsgD regulator. The entire *csg* gene cluster from *S.* Typhimurium was also cloned into *S*. Typhi, but again, despite introduction of a fully functional *csg* gene cluster from *S.* Typhimurium, curli were still not detected in *S.* Typhi. Thus, in addition to intrinsic genomic differences in the *csg* gene cluster that have resulted in production of a modified CsgD protein, *S*. Typhi has likely undergone other changes independent of the *csg* gene cluster that have led to distinctive regulation of *csg* genes compared to other *Salmonella* serovars*.*

## Introduction

*Salmonella enterica* serovar Typhi (*S.* Typhi) is a human-specific pathogen that causes a systemic disease known as typhoid fever. Typhoid fever affects an estimated 11–21 million people yearly and causes 128,000–161,000 deaths^1^. Typhoid fever has become a global public health concern as many endemic areas are facing the emergence of multiresistant strains^2,3^. Among recovered patients, 2–5% become chronic carriers^4^, that may develop due to colonization and biofilm formation in the gallbladder^5–7^. These chronic carriers currently represent the only known reservoir, and mechanisms controlling biofilm formation by *S.* Typhi are not well characterized. On the other hand, *Salmonella enterica* serovar Typhimurium (*S.* Typhimurium) is a non-typhoidal *Salmonella* and one of the main causes of human foodborne gastroenteritis. *S.* Typhimurium can colonize a large variety of hosts ranging from mammals to birds^8,9^. The host range and diseases caused by these two serovars are distinct although *S.* Typhimurium and *S.* Typhi share 89% of their genomes^10^. Among the genomic differences, *S.* Typhi contains more than 200 pseudogenes and 600 Typhi-specific genes compared to *S.* Typhimurium^10–15^. While the specific components and mechanisms underlying biofilm formation by *S.* Typhi are mostly unknown, *S.* Typhimurium biofilm includes cellulose, the BapA protein and curli fimbriae^16–20^. Curli are amyloid fibrils produced by certain bacteria such as *E. coli* and *S.* Typhimurium. Curli can mediate initiation of biofilm formation^18,20^. However, the role of curli in *S.* Typhi is still poorly understood. Curli are encoded by genes present in the two divergent operons, *csgBAC* and *csgDEFG*. CsgB is the nucleator mediating polymerisation of CsgA major structural subunits^21,22^. CsgC may help prevent intracellular polymerization of CsgA^23^. CsgG is a porin-like protein involved in secretion of extracellular curli components (CsgB, CsgA and CsgF) and has a chaperone-like function that promotes intracellular stabilization of CsgA and CsgB^20,21,24,25^. CsgF is an assembly protein which can localize CsgB on the bacterial surface^26^. CsgE is an accessory protein with a chaperone-like function that modulates which components can be secreted by the porin-like CsgG protein^23^. Finally, CsgD is a positive regulator belonging to the UhpA/FixJ/LuxR family, and is a DNA-binding protein. It is the main activating regulator of *csgBAC*^27^*.* In addition to regulation of curli-encoding genes, CsgD also plays a role in regulation of the O-antigen capsule, c-di-GMP, RpoS stress response and other biofilm components including regulation of BapA and cellulose synthesis in *S.* Typhimurium and *E. coli*^17,21,28,29^. Curli production is also controlled by several regulators that respond to different environmental stimuli such as temperature, oxygen, and osmolarity^21,30^. Under laboratory conditions, it was shown that the expression of curli by *S.* Typhimurium was highest after growth at 30 °C in lysogeny broth (LB) without salt or yeast extract, also called tryptone medium^31^. On Tryptone agar medium with Congo red dye, colonies are red, dry, and rough (rdar), corresponding to colonies that are producing both curli and cellulose. However, under the same growth conditions, *S.* Typhi colonies are smooth and white (saw), suggesting a lack of cellulose and/or curli expression^19,31^.

Thus, based on these results and on the fact that *S.* Typhi and *S.* Typhimurium have different pathogenic lifestyles, we hypothesized that in *S.* Typhi curli fimbriae are differently regulated compared to *S.* Typhimurium. The level of expression from the divergent promoters encoding each of the curli operons *csgBAC* and *csgDEFG* were compared between *S.* Typhi and *S.* Typhimurium under various growth conditions including different temperatures and oxygen levels. The role of CsgD on *csgB* expression was also investigated by determining DNA-binding to the *csgB* promoter region. *S.* Typhi *csgD* and curli gene cluster were replaced by the *S.* Typhimurium allele to investigate the impact of genetic polymorphisms on curli regulation.

## Results

### *csgB* expression profile

**Figure 1:**
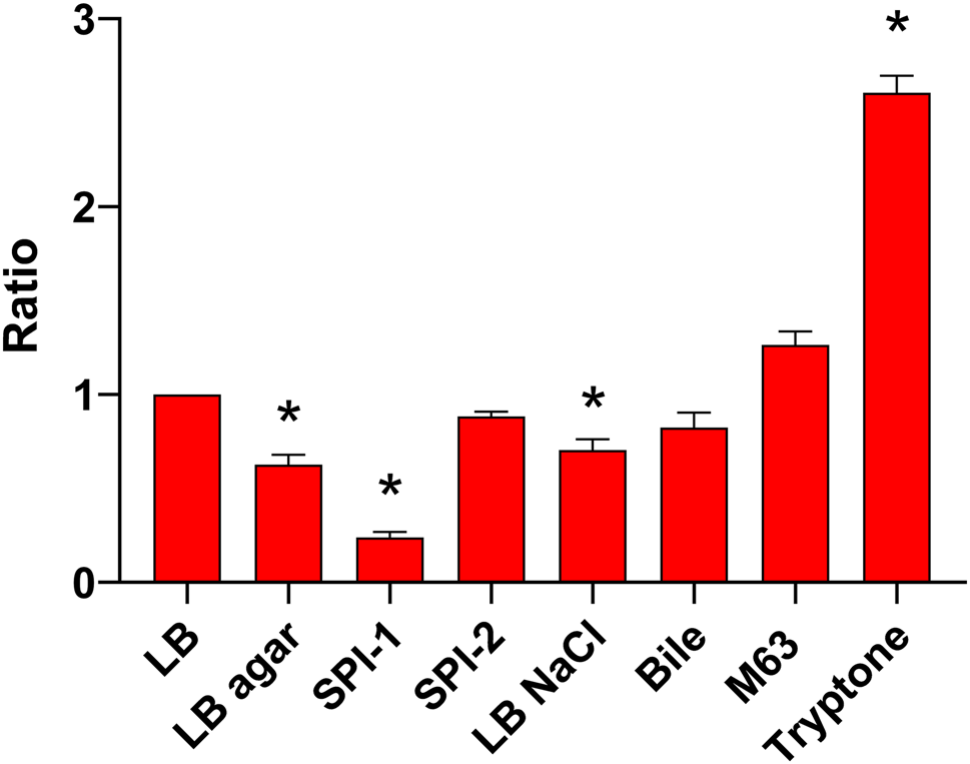
*S.* Typhi *csgB* expression in different growth media. *csgB* expression of WT *S.* Typhi was evaluated by β-galactosidase assays using p*csgB*_*S*Ty_-*lacZ* fusion vector. Expression is presented as a ratio comparing various media to expression levels after growth in LB. All cultures were incubated at 37 °C overnight. The experiments were repeated at least 3 times with 2 technical replicates. The negative control, WT *S.* Typhi transformed with pRS415 empty vector did not present significant expression level in all studied conditions. *p < 0.001.

Although curli expression is well characterized in *S.* Typhimurium, the optimal laboratory expression conditions in *S.* Typhi remain unclear. Thus, curli gene expression in *S.* Typhi (*S*Ty) was evaluated in different media using a *lacZ* reporter gene (p*csgB*_*S*Ty_-*lacZ*) and was compared with expression in standard LB (Fig. 1). *csgB* expression varied and was significantly lower on solid medium, under SPI-1 inducing conditions, and with 0.3 M NaCl, whereas the highest expression was in tryptone medium. These results suggested that *S.* Typhi *csgB* expression was regulated by differences in nutrient and oxygen levels. Tryptone medium was selected to further investigate *csg* expression.

**Figure 2:**
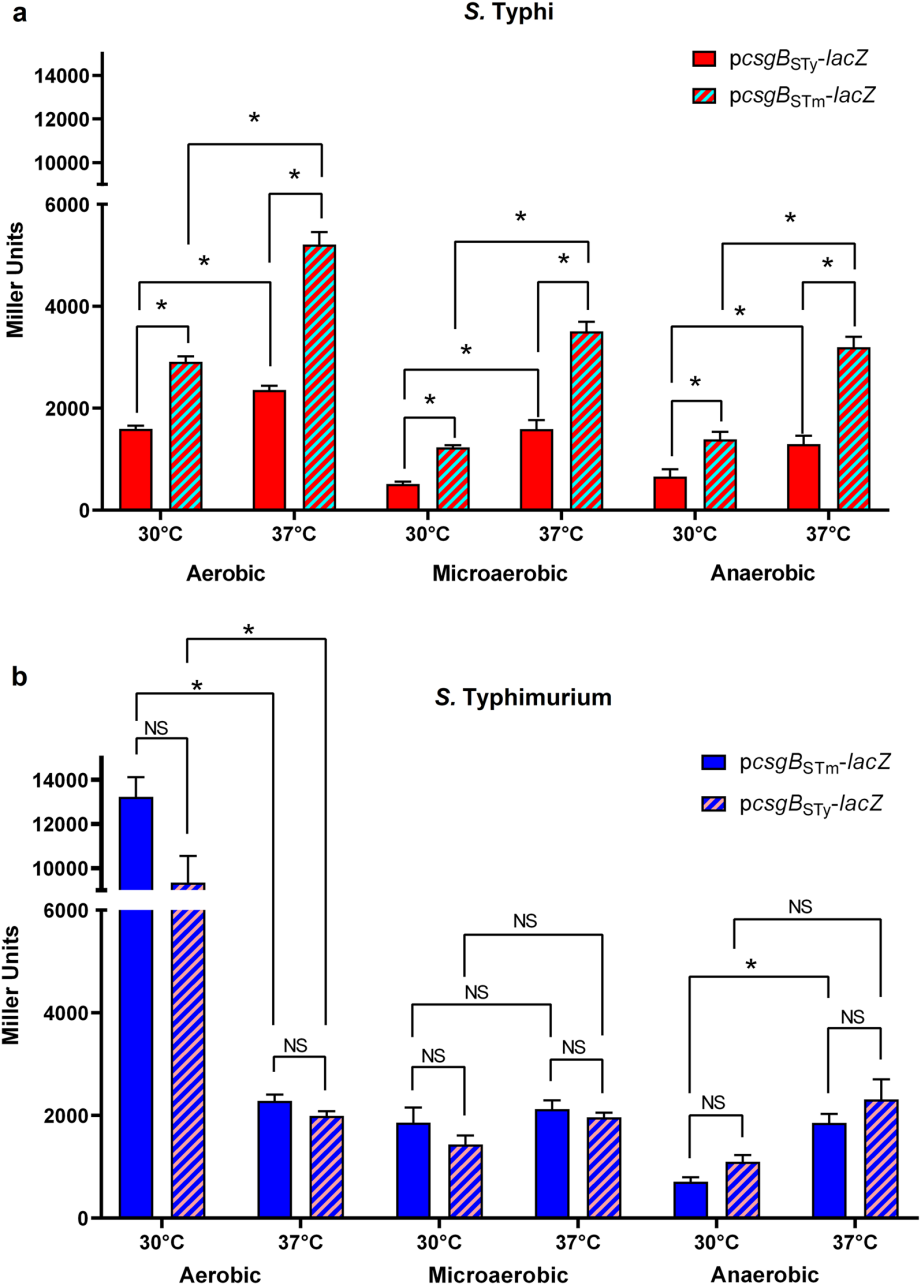
Effect of temperature and oxygen on *csgB* expression in *S.* Typhi and *S.* Typhimurium. *csgB* expression was evaluated by β-galactosidase assays in aerobic, microaerobic and anaerobic conditions, at 30 °C and 37 °C. (**a**) *csgB* expression in *S.* Typhi: p*csgB*_*S*Ty_-*lacZ* is in solid red and p*csgB*_*S*Tm_-*lacZ* is in red and light blue stripes. (**b**) *csgB* expression in *S*. Typhimurium: p*csgB*_*S*Tm_-*lacZ* is in solid blue and p*csgB*_*S*Ty_-*lacZ* is in blue and light red stripes. The experiments were performed at least 3 times with two technical replicates. The negative controls, WT *S.* Typhi and WT *S.* Typhimurium transformed with pRS415 empty vector did not present significant expression level in all studied conditions. *p < 0.01.

Impacts of temperature and oxygen levels on *csgB* expression were investigated using the *S.* Typhi (*S*Ty) p*csgB*_*S*Ty_-*lacZ* reporter (Fig. 2A) and the *S.* Typhimurium (*S*Tm) p*csgB*_*S*Tm_-*lacZ* reporter (Fig. 2B). In *S.* Typhi, *csgB* was always significantly more expressed at 37 °C, regardless of the oxygen level (Fig. 2A, red). While, the highest expression was under aerobic growth, *csgB* expression dropped under microaerobic and anaerobic environments, at both 30 °C and 37 °C, without changing expression pattern. In *S.* Typhimurium, the highest level of *csgB* was observed at 30 °C under aerobic conditions and was reduced at least sixfold at 37 °C. While *csgB* expression was reduced with a lower level of oxygen at 30 °C, the expression at 37 °C remained similar in all tested oxygen levels (Fig. 2B, blue). Overall, temperature and oxygen seem to impact *csgB* expression differently between serovars.

Knowing that there are polymorphisms within the genomes of these serovars^10,15,32^, the influence of the genetic background on *csgB* expression was investigated using the *S.* Typhimurium promoter (p*csgB*_*S*Tm_-*lacZ*) in *S.* Typhi (Fig. 2A, stripes) and the *S.* Typhi promoter (p*csgB*_*S*Ty_-*lacZ*) in *S.* Typhimurium (Fig. 2B, stripes). In all studied conditions, *csgB* expression in *S.* Typhi was 2 to 2.5-fold higher when expressing p*csgB*_*S*Tm_-*lacZ*. Interestingly, the expression patterns were still conserved, with the highest expression at 37 °C and no major oxygen effects on the expression pattern. When *S.* Typhimurium expressed the *S.* Typhi *csgB* promoter (p*csgB*_*S*Ty_-*lacZ*), no significant changes were observed compared with the *S.* Typhimurium *csgB* promoter. Thus, it was suspected that the genetic background might play a role in *csgB* regulatory differences between *S.* Typhi and *S.* Typhimurium.

### *csgD* expression profiles

**Figure 3:**
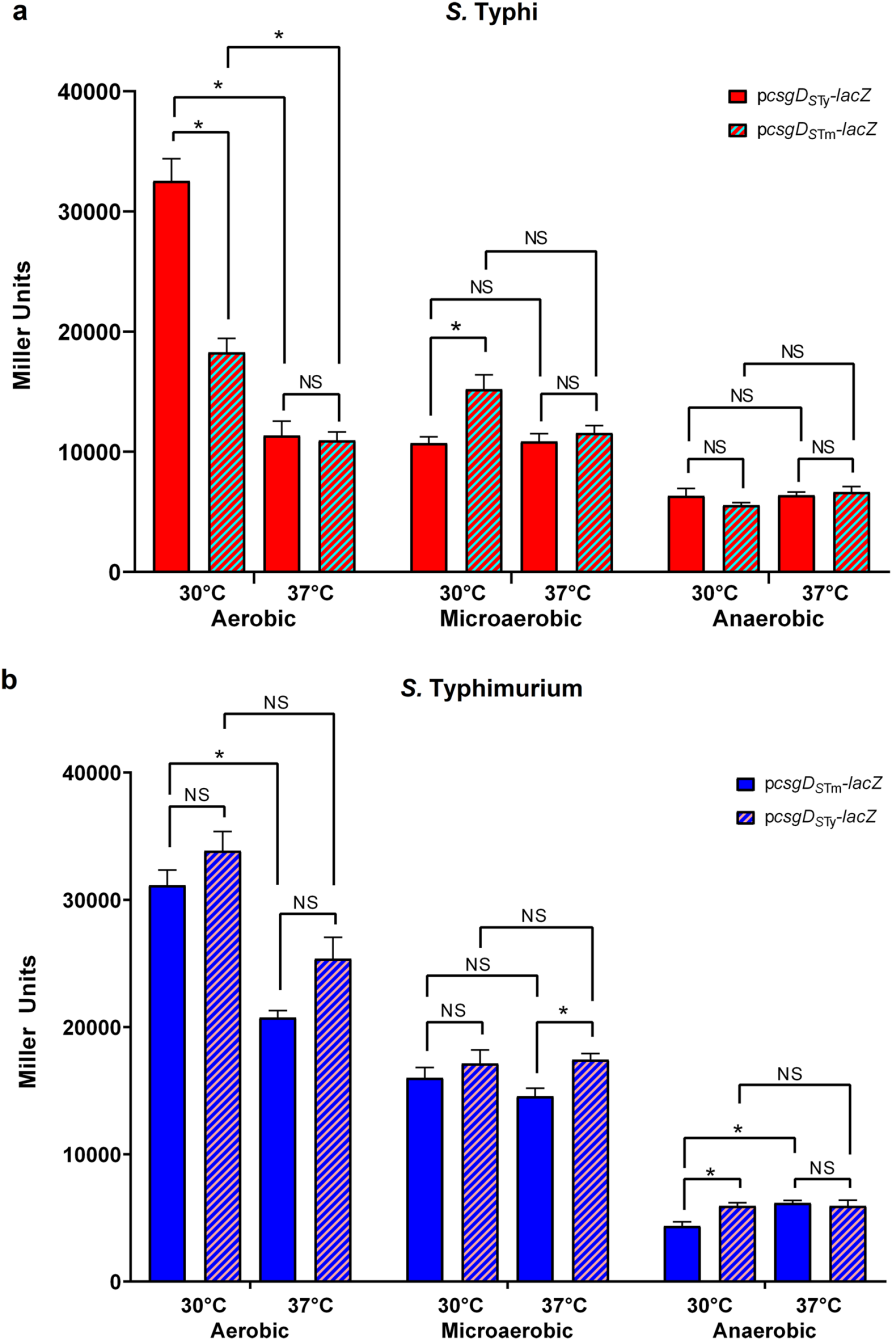
Effect of temperature and oxygen on *csgD* expression in *S.* Typhi and *S.* Typhimurium. *csgD* expression was evaluated by β-galactosidase assay in aerobic, microaerobic and anaerobic conditions at 30 °C and 37 °C. (**a**) *csgD* expression in *S.* Typhi: p*csgD*_*S*Ty_-*lacZ* is in solid red and p*csgD*_*S*Tm_-*lacZ* is in red and light blue stripes. (**b**) *csgD* expression in *S.* Typhimurium: p*csgD*_*S*Tm_-*lacZ* is in blue and p*csgD*_*S*Ty_-*lacZ* is in blue/light red stripes. The experiments were repeated at least 3 times with 2 technical replicates each. The negative controls, WT *S.* Typhi and WT *S.* Typhimurium transformed with pRS415 empty vector did not present significant expression level in all studied conditions. *p < 0.01.

CsgD is the main activator of the *csgBAC* operon^27^. Since the *csgB* expression profile differs between *S.* Typhi and *S.* Typhimurium, we evaluated the *csgD* expression profile under the same conditions*.* Surprisingly, in *S.* Typhi, the highest expression of p*csgD*_*S*Ty_*-lacZ* was at 30 °C under aerobic conditions, and significantly decreased when oxygen was less present (Fig. 3A, red). This pattern was different from what was observed for *csgB* expression (Fig. 2A, red) and was more similar to *S.* Typhimurium *csgB* expression (Fig. 2B, blue). In *S.* Typhimurium, the highest level of *csgD* was observed at 30 °C under aerobic conditions at a similar level to *S.* Typhi (Fig. 3A, red). Expression decreased with a lowest level in the absence of oxygen, at both 30 °C and 37 °C (Fig. 3B, blue).

Then, the influence of the genetic background was also investigated, by using the *csgD* promoter from *S.* Typhimurium (p*csgD*_*S*Tm_-*lacZ*) in *S.* Typhi (Fig. 3A, stripes) and the promoter from *S.* Typhi (p*csgD*_*S*Ty_-*lacZ*) in *S.* Typhimurium (Fig. 3B, stripes). When the p*csgD*_*S*Tm_-*lacZ* expression was evaluated in *S.* Typhi, a significant decrease of expression was observed in aerobic conditions at 30 °C*,* while a significant increase was observed in microaerobic conditions and no change in anaerobic conditions. No difference was observed at 37 °C, regardless of the oxygen level. When p*csgD*_*S*Ty_-*lacZ* was expressed in *S.* Typhimurium, the expression profiles were similar and conserved the same tendency, which was a decrease in expression with lower levels of oxygen, even if slightly higher in some conditions (Fig. 3B, stripes).

### CsgD binding capacity

The *csgD* expression profiles were similar for both *S.* Typhi and *S.* Typhimurium strains whereas *csgB* expression, which is known to be regulated by the CsgD regulator, differed considerably. We therefore investigated the binding capacity of CsgD proteins to the *csgB* promotor regions of each of these serovars. The DNA sequence of the *csgB* promotor regions in both *S.* Typhi and *S.* Typhimurium strains is identical.

**Figure 4:**
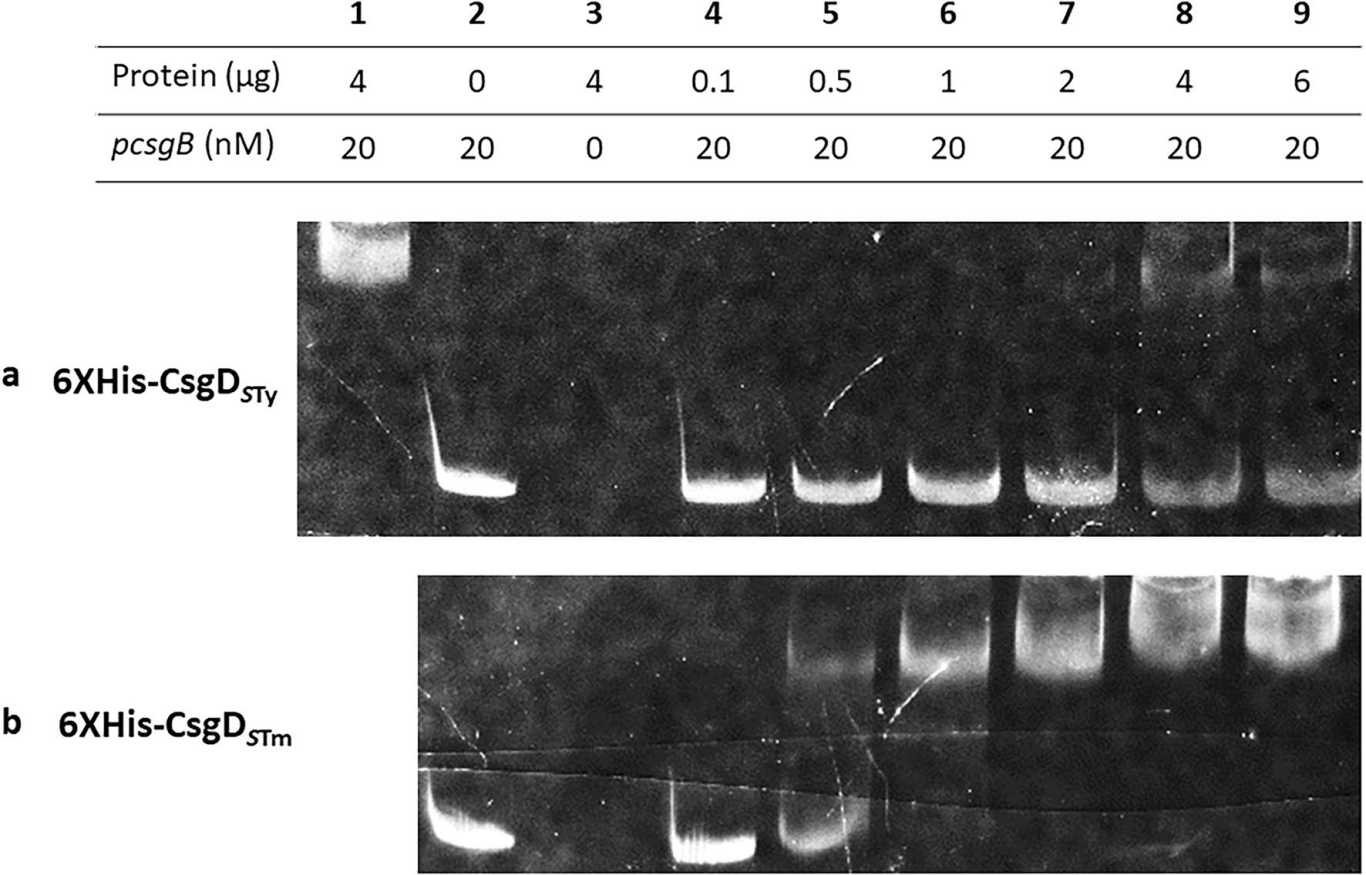
Capacity of CsgD proteins to bind the *csgB* promoter*.* (**a**) *S.* Typhi purified 6XHis-CsgD_*S*Ty_ and (**b**) *S.* Typhimurium purified 6XHis-CsgD_*S*Tm_ serving as a control. DNA probe P*csgB* consists of the *csgB* promoter region fused with fluorescein FAM. The promoter sequence is identical between *S.* Typhi and *S.* Typhimurium. (1) Control well using 6XHis-CsgD_*S*Tm_ and the DNA probe. (2) Control well with DNA probe only. (3) Control well with purified proteins only at 4 μg. (4–10) Increasing concentrations of 6XHis-CsgD from 0.1 to 8 µg with DNA probe.

Interestingly, CsgD of *S.* Typhi lacks the last 8 amino acids of the *S.* Typhimurium CsgD protein due to a single-nucleotide polymorphism (SNP) that introduces a premature stop codon^15,32^. It is unknown whether the truncated CsgD protein present in *S.* Typhi is functional^32^. Moreover, these missing amino acids are located in the DNA binding region of the regulator^33^. To evaluate the capacity of *S.* Typhi CsgD to bind to *csgB* promoter region, an EMSA (electrophoretic motility shift assay) was performed using purified *S.* Typhi 6XHis-CsgD (6XHis-CsgD_*S*Ty_) with a DNA probe (P*csgB*) (Fig. 4)*. S.* Typhimurium 6XHis-CsgD (6XHis-CsgD_*S*Tm_) was used as a control. 6XHis-CsgD_*S*Ty_ binding to the *csgB* promoter region was reduced when compared to CsgD of *S.* Typhimurium. For 6XHis-CsgD_*S*Ty_, a partial shift only occurred when 2 μg of protein were present. By contrast, 6XHis-CsgD_*S*Tm_ binding to the *csgB* promoter region began with 0.5 μg of protein and the band shift was complete with 1 μg of protein. These results demonstrate that the CsgD protein of *S.* Typhi has a lower DNA-binding capacity to the *csgB* promotor region when compared to CsgD_*S*Tm_.

### Effect of genomic substitution of *csgD* alleles in *Salmonella* Typhi and Typhimurium serovars

**Figure 5:**
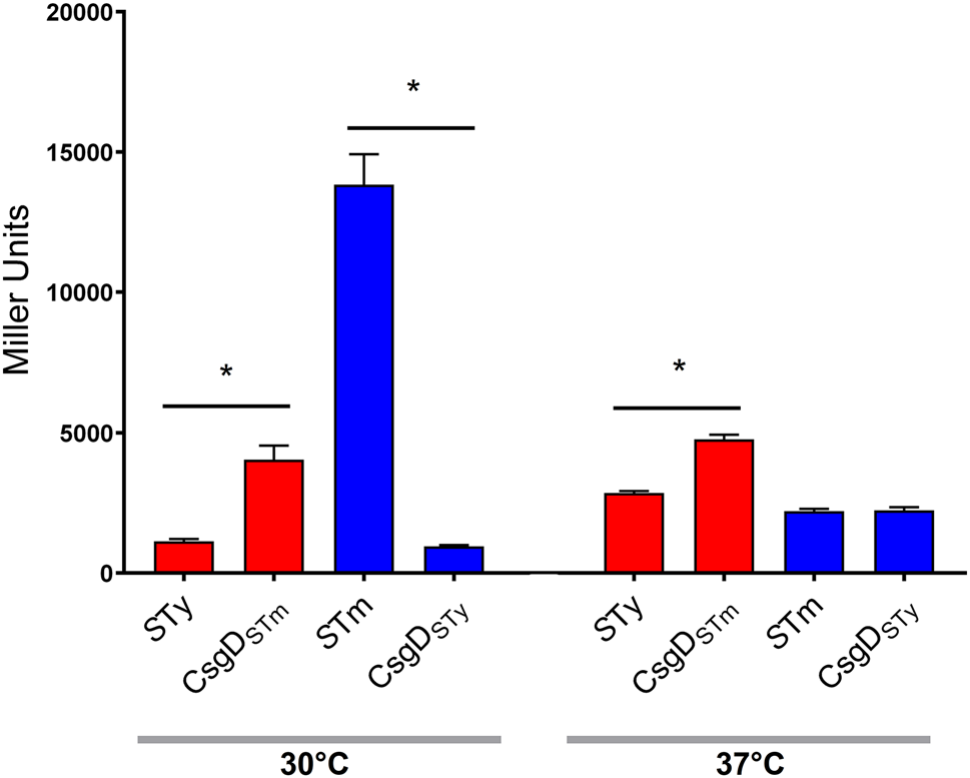
Effect of substituting *csgD* alleles in *S.* Typhi and *S.* Typhimurium on *csgB* expression. Expression of *csgB* in Miller units at 30 °C and 37 °C was evaluated by β-galactosidase Assay. Expression of *csgB* of *S.* Typhi in *S.* Typhi WT (*S*Ty) and in *S.* Typhi expressing *S.* Typhimurium *csgD* (CsgD_*S*Tm_) are presented in red. Expression of *S.* Typhimurium *csgB* in *S.* Typhimurium WT (*S*Tm) and in *S.* Typhimurium expressing *S.* Typhi *csgD* (CsgD_*S*Ty_) are presented in blue. The experiment was repeated at least 3 times with 2 biological replicates each. The negative controls, WT *S.* Typhi and WT *S.* Typhimurium transformed with pRS415 empty vector did not present significant expression level in all studied conditions. *p < 0.0001.

Knowing that CsgD_*S*Ty_ demonstrated a reduced DNA-binding capacity, we investigated the outcome of replacing the endogenous *csgD* gene in *S.* Typhi and *S.* Typhimurium with a heterogenous allele from the other serovar by allelic exchange in the genome. We then evaluated the effect of introduction of the different *csgD* alleles on *csgB* expression using the p*csgB*_*S*Ty_-*lacZ* or p*csgB*_*S*Tm_-*lacZ* fusion vectors (Fig. 5). As the greatest difference in *csgB* expression between *S.* Typhi and *S.* Typhimurium was observed during growth in aerobic conditions, this growth condition was selected for these experiments. In *S.* Typhi, introduction of the CsgD_*S*Tm_ allele increased *csgB* expression by fourfold at 30 °C and by 1.5-fold at 37 °C. However, this increase in expression still did not reach the same level as observed in WT *S.* Typhimurium. Interestingly, when grown at 30 °C, *csgB* expression decreased drastically by 14-fold in *S.* Typhimurium when the native *csgD* gene was replaced by *csgD*_*S*Ty_. By contrast, when grown at 37 °C, substitution of the native CsgD_*S*Tm_ with CsgD_*S*Ty_ in *S.* Typhimurium did not change *csgB* expression levels. These results suggest that the CsgD from *S.* Typhi is less able to activate *csgB* gene expression especially at 30 °C. However, in *S.* Typhi it is likely that the decreased activity of CsgD_*S*Ty_ alone is not the only factor contributing to differences in curli gene expression.

**Figure 6:**
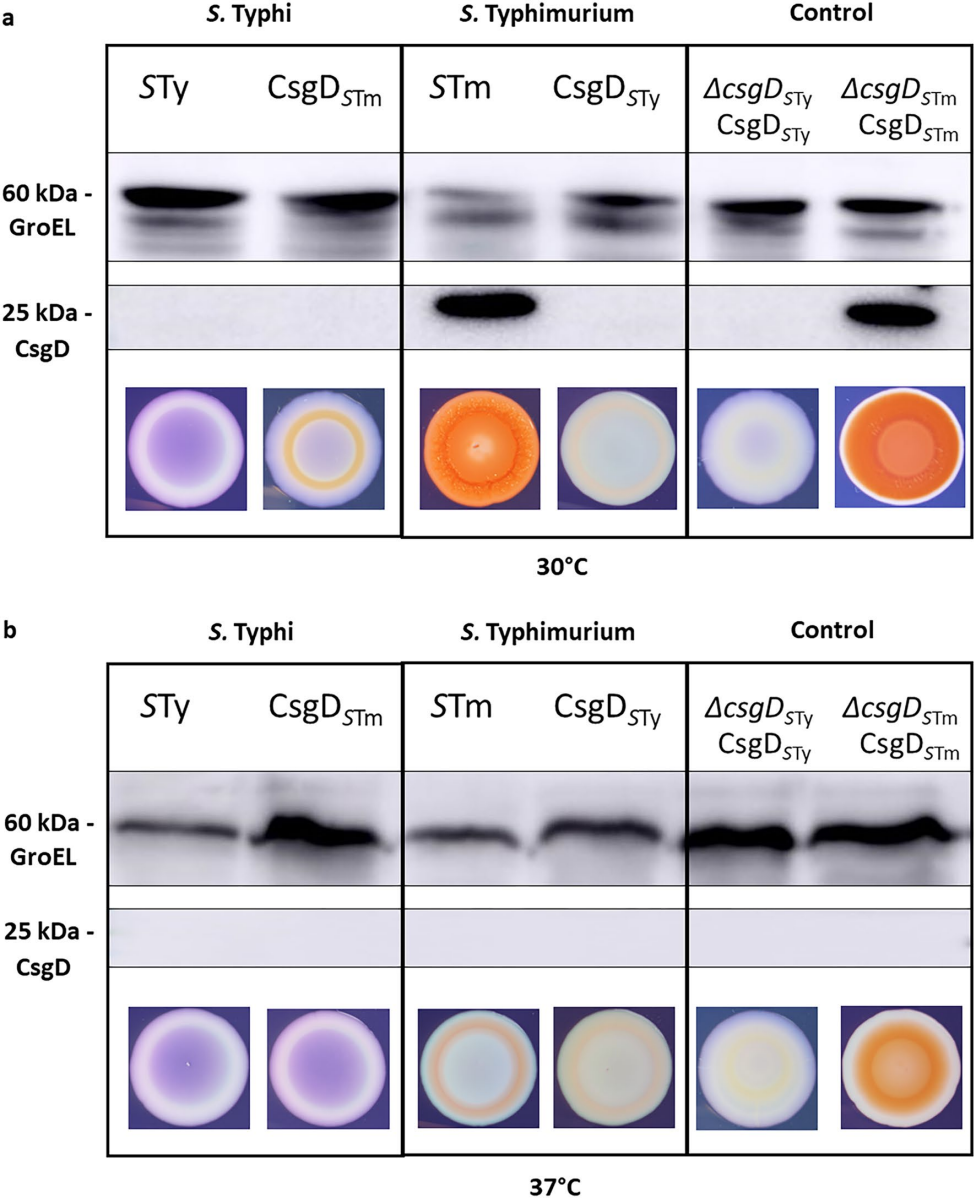
Impact of substituting *csgD* alleles on colonial morphology and production of CsgD protein. Detection of CsgD by Western Blot was performed on *S.* Typhi expressing *csgD* of *S.* Typhimurium (CsgD_*S*Tm_) and *S.* Typhimurium expressing *csgD* of *S.* Typhi (CsgD_*S*Ty_) and compared with wild-type protein expression—*S.* Typhi (*S*Ty) and *S.* Typhimurium (*S*Tm). Complementation controls were also tested—*S.* Typhi mutant ∆*csgD*_*S*Ty_ complemented with *csgD*_*S*Ty_ (∆*csgD*_*S*Ty_::*csgD*_*S*Ty_) and *S.* Typhimurium mutant ∆*csgD*_*S*Tm_ complemented with *csgD*_*S*Tm_ (∆*csgD*_*S*Tm_::*csgD*_STm_). All strains were grown in tryptone medium at (**a**) 30 °C and (**b**) 37 °C. Upper panels present detection of GroEL (~ 60 kDa) and lower panels present detection of CsgD (~ 25 kDa) by Western blot. Original blots are presented in Supplementary Fig. S3. Colonial morphology after 72 h was also evaluated by using Congo Red Plate Assay.

As the *csgB* expression levels were modified after substitution of the different *csgD* alleles, CsgD production was evaluated by Western Blot in these strains at 30 °C (Fig. 6A) and 37 °C (Fig. 6B). Surprisingly, despite the increase in *csgB* expression observed in *S.* Typhi containing *csgD* from *S.* Typhimurium (Fig. 5), no CsgD_*S*Tm_ was detected in *S.* Typhi at either 30 °C or 37 °C. On Congo red agar, a slight phenotypic change was observed with the appearance of a red ring within the colony at 30 °C but this was absent at 37 °C. For *S.* Typhimurium strains, only the WT and the control strain ∆*csgD*_*S*Tm_::*csg*D_*S*Tm_ (CsgD_*S*Tm_ reintroduced to complement the ∆*csgD*_*S*Tm_ mutant) at 30 °C showed production of a CsgD-specific band. Introduction of *csgD*_*S*Ty_ in *S.* Typhimurium caused loss of the typical rdar phenotype for a saw phenotype and no CsgD was detected by Western Blot (Fig. 6).

The previous set of experiments indicated that in *S.* Typhi, neither of the *csgD* alleles were able to produce a detectable protein, at either 30 °C or 37 °C. Further, when the *csgD*_*S*Ty_ allele was introduced into the *S.* Typhimurium strain background, no CsgD protein could be detected. These results suggest that CsgD production in *S*. Typhi may be silenced or nonfunctional. Further, in *S.* Typhimurium, lack of detection of any CsgD_*S*Ty_ protein could be due either instability of the truncated protein or differences in the *csgDEFG* promoter region in *S*. Typhimurium. In these strains, although the heterogenous copies of *csgD* alleles were introduced, the endogenous promoter regions were maintained.

**Figure 7:**
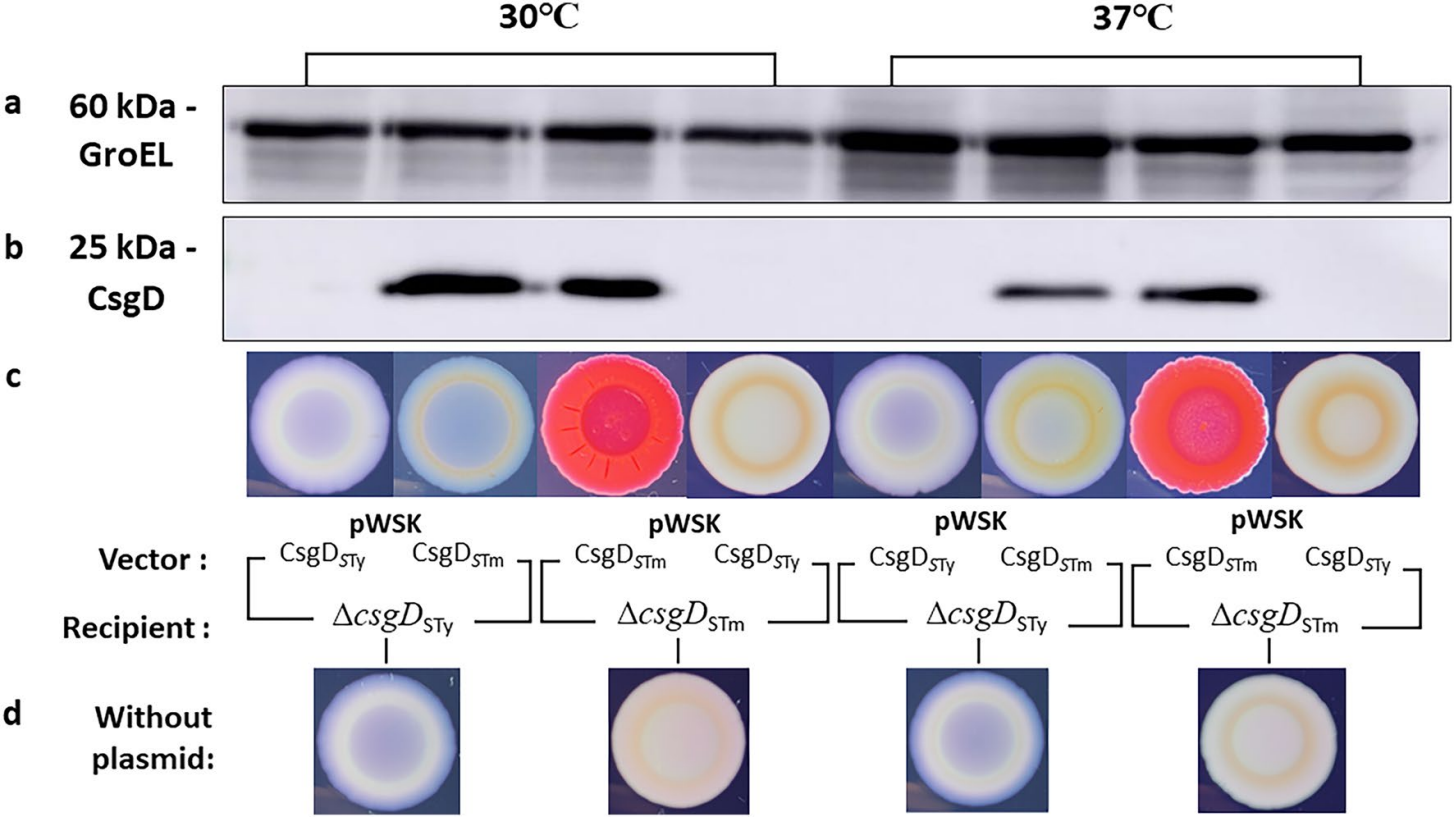
Introduction of *csgD*_*S*Ty_ or *csgD*_*S*Tm_ alleles cloned on a low-copy vector to Δ*csgD* mutants. Expression of CsgD was evaluated by Western blot of complemented strains grown in tryptone medium at 30 °C and 37 °C. (**a**) GroEL detection (~ 60 kDa). (**b**) CsgD detection (~ 25 kDa). Original blots are presented in Supplementary Fig. S3. (**c**) Congo Red Plate Assay of complemented and substituted strains of *S.* Typhi (∆*csgD*_*S*Ty_) or *S.* Typhimurium (∆*csgD*_*S*Tm_) with either pWSK-*csgD*_*S*Ty_ or pWSK-*csgD*_*S*Tm_. (**d**) Congo Red Plate Assay on ∆*csgD* strains without plasmid as controls. Colonial morphology was evaluated after 72 h of incubation.

Thus, to verify if the inability of detecting CsgD_*S*Tm_ in *S.* Typhi was due to possible protein degradation or expression inhibition, we complemented the *csgD* mutants (∆*csgD*_*S*Ty_ and ∆*csgD*_*S*Tm_) with *csgD* from each serovar expressed from a low-copy plasmid (pWSK-*csgD*_*S*Ty_ and pWSK-*csgD*_*S*Tm_). Production of CsgD at 30 °C and 37 °C was evaluated by Western blot (Fig. 7). The control *∆csgD*_*S*Tm_ complemented with pWSK-*csgD*_*S*Tm_ has a band for CsgD detection and the rdar phenotype was restored, but again, no CsgD was detected when CsgD of *S.* Typhi (pWSK-*csgD*_*S*Ty_) was cloned into *S.* Typhimurium and the rdar phenotype was not restored. For *S.* Typhi, results were different then with the genomic substitutions of *csgD* alleles. For *csgD*_*S*Tm_ expressed from the vector promoter, CsgD was detected by Western Blot in *S.* Typhi (∆*csgD*_*S*Ty_ pWSK-*csgD*_*S*Tm_) at both temperatures, but at lower level after growth at 37 °C. However, on Congo red agar, colonial morphology remained white and smooth with a red ring as in Fig. 6A.

### Introduction of a complete functional *csg* gene cluster to *S.* Typhi and *S.* Typhimurium strains

**Figure 8:**
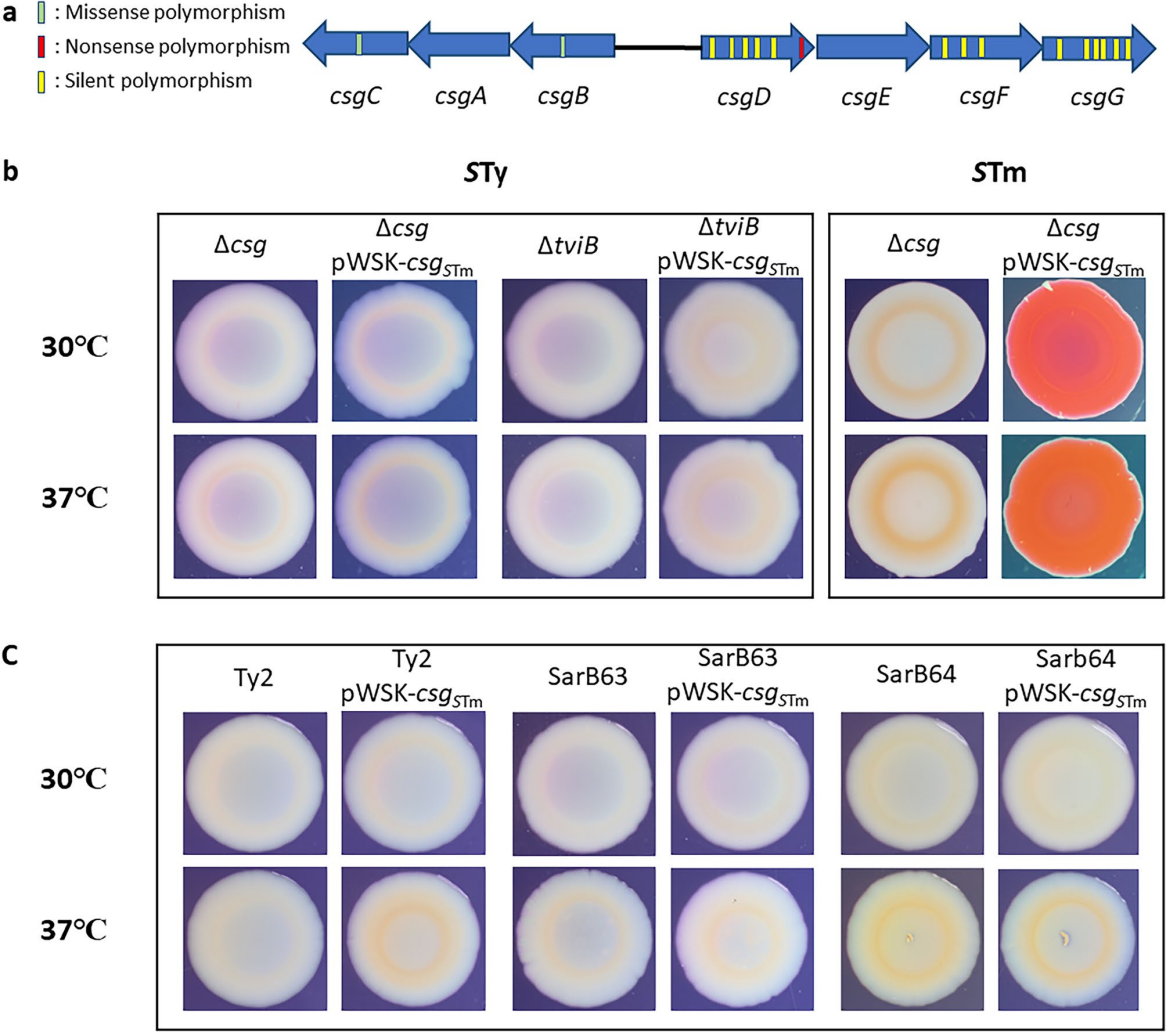
Complementation of curli operons. (**a**) Presence of single-nucleotide polymorphisms (SNPs) in the *S.* Typhi *csg* gene cluster. Missense SNPs are presented in green, nonsense SNPs in red and silent SNPs are in yellow. The nucleotides and amino acid sequences are presented in Fig. S1. (**b**) Congo Red Plate Assay at 30 °C or 37 °C of *S.* Typhi (*S*Ty) and *S.* Typhimurium (*S*Tm) curli ∆*csg* (∆*csgBACDEFG*) mutant and ∆*csg* complemented with a complete *csg* gene cluster from *S.* Typhimurium present on vector pWSK-*csg*_*S*Tm_ (pWSK-*csgBACDEFG*_*S*Tm_). A ∆*tviB* mutant and ∆*tviB::*pWSK-*csg*_*S*Tm_ containing a functional Csg system from *S.* Typhimurium were used as controls to determine if the Vi capsule could interfere with Congo Red binding and colonial morphology. (**c**) Congo Red Plate Assay at 30 °C or 37 °C of other *S.* Typhi strains: SarB63 and SarB64.

Since there were important differences in the *csgD* gene between *S.* Typhi and *S.* Typhimurium, we investigated whether other genes in the *csg* clusters contained polymorphisms that could affect production of curli. We compared *S.* Typhi and *S.* Typhimurium *csg* gene clusters and found multiple SNPs (silent, missense and nonsense SNPs) in the *S.* Typhi *csg* gene cluster (Fig. 8A and Supplementary Fig. S1). Due to the presence of multiple SNPs in different *csg* genes in *S.* Typhi, we introduced the entire *csg* gene cluster (*csgABCDEFG*) of *S.* Typhimurium into a *csg* mutant ∆*csg*_*S*Ty_ (∆*csgBACDEFG*) of *S.* Typhi using a low-copy pWSK plasmid (pWSK-*csg*_*S*Tm_) and evaluated the phenotype by Congo Red Plate Assay (Fig. 8B). pWSK-*csg*_*S*Tm_ was also electroporated into a ∆*csg*_*S*Tm_ mutant of *S.* Typhimurium as a control. In the *S.* Typhimurium ∆*csg* strain, introduction of the *csg* gene clusters resulted in a regain of the rdar phenotype, confirming that production of curli can be complemented by this plasmid. However, when this same pWSK-*csg*_*S*Tm_ plasmid was cloned into the *S.* Typhi ∆*csg* mutant, no changes in colonial morphology were observed and *S.* Typhi colonies retained a saw phenotype. These results indicate that in addition to polymorphisms in the *csg* gene cluster that may alter *csg* gene expression and curli production, *S.* Typhi also likely contains other polymorphisms external to *csg* that reduce or inhibit *csg* gene expression or curli production under the growth conditions used in this study. To ensure that the lack of colonial morphology changes was not due to the presence of the Vi capsule- as extracellular components such as polysaccharide might interfere with Congo Red binding^34^, we also investigated colonial morphology in a Vi capsule-negative ∆*tviB* mutant of *S.* Typhi with pWSK-*csg*_*S*Tm_. Regardless, loss of the Vi capsule did not alter the saw colony phenotype. Lastly, we investigated whether this lack of rdar phenotype was strain specific by introducing pWSK-*csg*_*S*Tm_ in different *S.* Typhi strains (Ty2, SarB63 and SarB64) (Fig. 8C). Again, no significant morphology change was observed suggesting a potential serovarspecific curli down regulation.

## Discussion

Curli are known to be an important component of biofilm formation by non-typhoidal *Salmonella enterica* serovars such as *S.* Typhimurium^18,20^. *S.* Typhi persistence in asymptomatic carrier hosts was demonstrated to involve biofilm formation in the gallbladder^5–7^. Expression and regulation of curli in *S.* Typhi are poorly understood. It was suggested that curli were non-functional in *S.* Typhi, as a white and smooth phenotype (saw) was observed on Congo red agar using standard growth conditions for the curli positive rdar (red, dry and rough) morphotype^21,31,32^. However, antibodies against curli components have been found in patients with typhoid fever^35^, suggesting a potential for curli production during typhoid fever infections.

By comparing media mimicking conditions encountered at different infection stages, we have determined that the highest level of expression of *csgB* in laboratory conditions was in tryptone medium, a low salt and low nutrient medium (Fig. 1). This medium is also known to promote curli production in *S.* Typhimurium^31^. Other growth conditions such as temperature and oxygen levels encountered during infection were also investigated and their effect on curli gene expression in *S.* Typhi was compared to a *S.* Typhimurium strain. The highest expression of *csgB* in *S.* Typhi was always at 37 °C (Fig. 2). This is in line with a potential for *csg* gene expression by *S.* Typhi in the human host. Expression of *csgB* in *S.* Typhimurium was sixfold higher at 30 °C than at 37 °C. This was only observed under aerobic conditions, while reducing oxygen levels decreased the effect of temperature. Temperature and oxygen levels seem to have an interdependent effect on *S.* Typhimurium. However, oxygen levels did not affect expression in *S.* Typhi. These results confirm that *csg* gene expression in *S.* Typhi does not follow the same regulation as *S.* Typhimurium. Thus, we investigated the role of the genetic background on curli expression by measuring expression of the *S.* Typhimurium *csgB* promoter including the intergenic region in *S.* Typhi (Fig. 2). About 2–2.5-fold higher expression was observed in all studied conditions and growth at 37 °C promoted the highest level of expression. However, strikingly, expression levels were distinct compared to those in *S.* Typhimurium. Based on these results, it was expected that in *S.* Typhimurium the *csgB* promoter of *S.* Typhi should have a decreased expression profile. Surprisingly*,* no difference in expression was observed in all the studied conditions. This further implies that regulation of curli in *S.* Typhi is different.

Many regulators are implicated in curli expression. Each one has various roles responding to environmental stress and culture conditions and temperature^20,21^. CsgD is the main regulator that activates curli expression. Thus, we evaluated its expression profile to compare it with *csgB* expression (Fig. 3)^27^. The most striking result was that both serovars presented similar expression profiles at 30 °C under aerobic conditions. Knowing that *csgB* expression was significantly different (Fig. 2), these results suggested that the potential expression differences between these serovars may be due to post-transcriptional regulation that implicates the CsgD regulator. It was previously reported that *csgD* expression was highest in microaerobic conditions in *S.* Typhimurium^21,30^. While our results propose that expression levels of *csgB* and *csgD* were higher in aerobic conditions. However, the growth conditions were different between these studies.

Since the expression level of *csgD* was similar between *S.* Typhi and *S.* Typhimurium but expression of *csgB* was lower in *S.* Typhi, we investigated the impact of CsgD regulation on *csgB* expression. CsgD is a member of the UhpA/FixJ/LuxR family with a DNA binding site in the C-terminal portion^27,33^. *S.* Typhi CsgD (CsgD_*S*Ty_) lacks the last 8 amino acids compared to the CsgD_*S*Tm_ protein due to a nonsense polymorphism^32^. Thus, we investigated if CsgD_STy_ binding to the *csgB* promoter is altered compared to CsgD_*S*Tm_ (Fig. 4). We found that CsgD_*S*Ty_ was only able to partially bind the *csgB* promoter region even at high concentration while CsgD_*S*Tm_ was able to effectively bind at lower protein concentrations. Our results demonstrated that CsgD_*S*Ty_ had reduced functionality and binding-capacity to the *csgB* promoter region. This might explain the significantly lower expression of *csgB* in *S.* Typhi compared to *S.* Typhimurium (Fig. 2). To confirm if this reduced binding profile alters curli regulation and expression, *csgD*_*S*Ty_ was integrated into the *S.* Typhimurium genome to substitute for the native *csgD*_*S*Tm_ open reading frame without altering the native promoter region. In this strain, *csgB* expression was drastically reduced (Fig. 5) and the rdar phenotype of *S.* Typhimurium on Congo Red agar was lost (Fig. 6). Interestingly, the CsgD_*S*Ty_ protein could not be detected by Western Blot despite growth conditions in *S.* Typhimurium that favour CsgD expression. The possibility of issues with antibody specificity were eliminated since the monoclonal anti-CsgD antibody also effectively detected the 6XHis-CsgD_*S*Ty_ protein (Supplementary Fig. S2). The absence of production of CsgD_*S*Ty_ suggests that the protein is degraded or not functional in *S.* Typhimurium. CsgD_*S*Ty_ in *S.* Typhi was also never detected under any conditions, and will be discussed below.

When the functional *csgD*_*S*Tm_ gene was introduced into *S.* Typhi, *csgB* expression did increase by 1.5- to 4-fold depending on the growth conditions. However, expression was still significantly lower than that observed in WT *S.* Typhimurium (Fig. 5). This suggests that the CsgD_*S*Ty_ was only one of a number of other factors leading to differences in *csg* gene expression between serovars. Other genomic polymorphisms outside of the *csg* gene cluster are likely to prevent high level expression of curli in *S.* Typhi under the growth conditions used in the study. Surprisingly, despite the increase of gene expression, no CsgD_*S*Tm_ protein was detected by Western Blot (Fig. 6). Since the *csgD* allele substitution experiments only replaced the open reading frames encoding the CsgD regulatory proteins, it is possible that differences in *csgDEFG* promoter regions could also affect expression of the *csg* gene clusters. We therefore evaluated the effect of *csgD*_*S*Tm_ expressed from its native promoter region in a low-copy plasmid to complement ∆*csgD* mutant to bypass this regulation. In this way, we were able to detect CsgD_*S*Tm_ in *S.* Typhi (Fig. 7). This result refutes the likelihood that in *S.* Typhi the CsgD_*S*Tm_ protein may be unstable or is degraded and confirms that in *S.* Typhi expression of *csgD* from the endogenous CsgD_STy_ promoter is inhibited under the growth conditions used in the laboratory. Despite the production of CsgD_STm_ from the low-copy plasmid in *S.* Typhi, this strain still maintained a saw phenotype on Congo Red Agar, indicating that other polymorphisms or modifications in the *S.* Typhi genome may further affect production of curli.

The nonsense SNP in the CsgD_*S*Ty_ sequence reduced DNA-binding efficiency of this regulatory protein, suggesting an underlying reason why curli gene expression may be altered in *S.* Typhi. However, as the rdar morphotype was not recovered after introduction of a functional CsgD_*S*Tm_ regulatory protein, it is possible that polymorphisms in other genes involved in curli expression or synthesis could also alter curli function in *S*. Typhi. In fact, analysis of the *S.* Typhi genome in the *csg* gene cluster identified several additional SNPs and most of the genes encoding curli assembly components present SNPs, with the majority being silent, although two are missense and only CsgD has a nonsense SNP (Fig. 8). To determine if a combination of modifications within the *csg* gene cluster in *S.* Typhi was potentially responsible for differences in curli gene expression or production in *S.* Typhi, we cloned the entire functional gene cluster from *S.* Typhimurium into a low-copy vector and introduced this plasmid into *S.* Typhi and *S.* Typhimurium ∆*csg* (∆*csgBACDEFG*) mutants. This pWSK-*csg*_*S*Tm_ plasmid was confirmed to be functional as complementation of the *S.* Typhimurium ∆*csg* mutant which had a saw colonial morphotype regained the rdar phenotype, that is associated with production of curli (Fig. 8). By contrast, the pWSK-*csg*_*S*Tm_ plasmid when introduced into the *S.* Typhi ∆*csg* mutant*,* did not alter the saw phenotype at either 30 °C or 37 °C. The lack of a rdar phenotype after introduction of the *csg*_*S*Tm_ gene cluster was also not due to presence of the Vi capsule which might interfere with the Congo Red Plate Assay. Congo Red dye could react differently with various cellular surface components^34,36^. Loss of the Vi capsule in a strain containing the plasmid encoding the *csg*_*S*Tm_ gene cluster remained white and smooth (Fig. 8B), as well as other Typhi strains like Ty2, SarB63 and SarB64 expressing *csg*_*S*Tm_ gene (Fig. 8C). Thus, this lack of morphology change seems to be serovar-specific.

Taken together, these results suggest that in *S.* Typhi, altered regulation or inhibition of curli production is multitiered and involves a missense SNP in CsgD that truncates the protein and reduces its DNA-binding capacity. In addition, there are likely additional as yet undefined modifications or polymorphisms beyond the *csg* gene cluster that lead to inhibition of curli production. Since the functional *csg* gene cluster from *S*. Typhimurium cloned into *S*. Typhi did not confer production of curli or the rdar colonial morphotype even under conditions favourable for curli production at 30 °C. Conditions that may promote expression of curli fimbriae in *S.* Typhi are still unknown as well as overall mechanisms underlying differences in its multitiered regulatory control.

Curli are known to promote adherence to intestinal epithelial cells and activate the immune system via toll-like receptor TLR-2^37,38^. TLR-2 also plays a role in PI3K mediated tight-junction barrier response which is a host innate immune mechanism to prevent bacterial dissemination. Hence, *S.* Typhimurium causes a local intestinal inflammation^39^. Contrary to non-typhoidal *Salmonella* infection, *S.* Typhi does not cause inflammation in the gut and it is able to bypass some innate immune defenses during infection^40,41^. Thus, in the intestine inhibition of curli expression may be critical for *S.* Typhi to prevent TLR-2 activation leading to increased intestinal epithelial barrier integrity. However, curli might nevertheless play a role at other stages of *S.* Typhi infection under specific conditions yet to be determined such as for biofilm production to promote persistence and the chronic carrier state. We are currently investigating potential alternative regulation mechanisms for curli expression in *S.* Typhi that may be distinct from regulation in most other *Salmonella enterica* serovars.

## Conclusions

Curli fimbriae encoded by the *csg* gene cluster have been characterized extensively in *E. coli* and other *Salmonella*. However, little is known specifically about curli gene expression in *Salmonella* Typhi. Here, we demonstrate that *S.* Typhi curli gene expression does not respond to the same environmental cues as the *csg* genes in *S.* Typhimurium. Importantly, it was demonstrated that the regulatory protein CsgD, which is the major regulator of curli production and biofilm formation in *S.* Typhimurium, has a reduced DNA-binding capacity to the *csgB* promoter, which controls expression of the curli subunit encoding gene. Further, introduction of a functional *csg* gene cluster cloned from *S.* Typhimurium was unable to promote production of curli in *S*. Typhi under conditions that are normally favourable for its production in *Salmonella* and *E. coli*. Taken together, results confirm that the regulatory mechanisms underlying curli gene regulation in *S.* Typhi are multitiered and include genomic polymorphism within the *csgD* gene and other as yet undefined modifications elsewhere in the genome. Results also suggest that if curli are produced by *S.* Typhi, expression is subject to regulatory cues that are distinct from those regulating curli in *Salmonella* Typhimurium and *E. coli*.

## Materials and methods

### Conditions and media used for bacterial growth

All bacterial strains and plasmids used are listed in Supplementary Table S1. All strains were routinely grown in lysogeny broth (LB) or LB agar at 37 °C overnight (18 h). When required, antibiotics or supplements were added at the following concentrations: 100 μg/mL for ampicillin, 34 μg/mL for chloramphenicol, 50 μg/mL for diaminopimelic acid (DAP) and 0.5 mM for IPTG.

### Cloning of curli promoters and β-galactosidase assays

Promotor regions of *csgBAC* (P*csgB*) and *csgDEFG* (P*csgD*) from *S.* Typhi (*S*Ty) ISP1820 and *S.* Typhimurium (*S*Tm) SL1344 were amplified by PCR using csg_prom_F_short_EcoRI/csg_prom_R_BamHI for P*csgB* and csgD_Prom_F_EcoRI/csgD_Prom_R_BamHI for promoter P*csgD* (Supplementary Table S2). The promoters were inserted in vector pRS415 to generate *lacZ* fusions using restriction enzymes (Anza) *Eco*RI and *Bam*HI, and T4 ligase (NEB). The resulting plasmid was transformed into WT ISP1820 and SL1344 by electroporation as described in O’Callaghan et al.^42^. Clones were confirmed by PCR and sequenced using respective promoter forward primer and the lacZ_alpha_R primer from the pRS415 vector. Empty vector (pRS415) was also electroporated in wild-type strains to serve as negative control for β-galactosidase assay.

After growth under various culture conditions, promoter expression was evaluated by β-galactosidase assay as described by Miller^43^. For *csgB* expression, various media were used to mimic different environments encountered during the infection process. For host invasion, SPI-1 was induced in LB with 0.3 M NaCl without agitation for low oxygenation^44,45^. To mimic intracellular conditions encountered in macrophages, SPI-2 was induced in low phosphate medium (LPM)^46^. For high osmolarity conditions, salt (0.3 M NaCl) was added to LB and incubated with agitation. M63 minimal medium, was used as a defined medium^47^. Effect of bile was tested using LB with 3% ox-bile (Sigma), as *S.* Typhi produces biofilm in gallbladders of asymptomatic chronic hosts^7,20^. LB without any salt or yeast extract also called tryptone medium, was also tested as it is the optimal medium for *S.* Typhimurium curli expression under laboratory conditions^31^. For determination of *csgB* and *csgD* expression levels, bacteria were grown at different temperatures. 37 °C was studied as *S.* Typhi is a human specific pathogen. Experiments were also performed at 30 °C, as it is the optimal temperature for curli expression in *S.* Typhimurium^31^. For oxygen impact on expression, bacteria were incubated with agitation for aerobic condition, without agitation for microaerobic conditions and using a GasPak system (MGC) for anaerobic growth. Experiments were done at least three times in duplicate. In all the studied conditions, no significant expression was observed for the negative control.

### Purification of 6XHis-CsgD protein and electrophoretic motility shift assay (EMSA)

High levels of ISP1820 and SL1344 CsgD were produced using the pET system (Novagen). The *csgD* gene of both serovars was cloned in expression vector pET14b by PCR using primers CsgD-F-NdeI/CsgD-R-BamHI and transformed in *E. coli* host strain BL21(DE3). To harvest 6XHis-CsgD, a preculture was performed overnight, then diluted 1:50 in 1 L of fresh LB. The culture was incubated until OD_600_ reached 0.5–0.6 followed by addition of 0.5 mM IPTG and induction at 25 °C overnight. Bacteria were then treated and lysed following as described in the pET System Manual (Novagen) to isolate the soluble fraction. 6XHis-CsgD protein was then purified by gravity affinity chromatography (Bio-RAD) using Ni-NTA resin (Thermo-Fisher).

To perform the EMSA, a fluorescent probe of the P*csgB* promoter region was synthesized by PCR amplification using a fluorescein fused primer curli-inter-F-FAM and curli-inter-R. The selected *csgB* promoter region sequences of *S.* Typhi ISP1820 and *S.* Typhimurium SL1344 are identical. Different concentrations of previously purified 6×His-CsgD with DNA probes were used. A 20 μL volume of each mixture was prepared in EMSA buffer (50 mM NaCl, 20 mM Tris pH 7.4 and 0.02% sodium azide). Every reaction was incubated for 40 min at 30 °C to allow protein binding before adding 5 μL of loading buffer (50 mM Tris pH7.4, 50 mM EDTA, 0.1% Bromophenol Blue and 12.5% sucrose). Migration of the reactions was performed using 5% native polyacrylamide gel containing 0.5×TAE buffer (20 mM Tris Base, 10 mM acetic acid, 0.5 mM EDTA) and 2% glycerol during 30 min at 150 V. Imaging of fluorescent probes was performed using a ChemiDoc imaging system (Bio-RAD) and Image Lab Software.

### Cloning, deletion and allelic exchange of *csgD*

Mutagenesis of *csgD* was performed by allelic exchange with suicide vector pMEG375 as described in Forest et al*.*^48^ using two pairs of primers, CsgD_F1/CsgD_R2 and CsgD_F3/CsgD_R4, targeting the upstream and downstream region of the gene. *csgD* markerless and nonpolar deletion was done in *S.* Typhi ISP1820 and *S.* Typhimurium SL1344.

For gene complementation by low-copy plasmid pWSK29*, csgD* from *S.* Typhi or *S.* Typhimurium was amplified by PCR using primer pair csgD_Prom_F_BamHI and csgD_R_XbaI. The generated insert was digested with BamHI and XbaI (Anza) and ligated with T4 ligase (NEB). The same procedure was used for cloning of the *csg* gene cluster with primer pair Csg_F and Csg_R. Plasmids were transformed into the following mutants and *Salmonella* strains: ∆*csgD*, ∆*csg* (∆*csgBACDEFG*), ∆*tviB,* wild type Ty2, SarB63 and SarB64.

Introduction of the heterogenous *csgD* gene from the other serovar was achieved by cloning the *csg* genes from *S.* Typhi ISP1820 or *S.* Typhimurium SL1344 by PCR using primer pair csgD_swap F/csgD_swap_R. The generated amplicons were inserted into suicide vector pMEG375 following the same procedure as the described for mutagenesis. Allelic exchange was performed using the Δ*csgD* mutant of *S.* Typhi ISP1820 and *S.* Typhimurium SL1344 as recipients. Each serovar received the heterogenous *csgD* from the other serovar. Complementation with the endogenous *csgD* gene was also performed as a control. All vectors were confirmed by PCR and sequencing.

### Congo red plate assay

Strains were grown overnight in LB with agitation and ampicillin when required. A volume of 3.5 μL of culture was plated on Congo Red Agar (1% tryptone, 1% agar, 40 μL/mL of Congo Red Dye, 0.8 μL/mL of Coomassie Blue) and ampicillin when required. Plates were incubated at 30 °C or 37 °C for 72 h. Pictures of colonies were taken using an Olympus DP22 digital camera and its software with Olympus SZ61 stereomicroscope.

### SDS-PAGE and western blot

Bacteria were grown in 50 mL of tryptone medium at 30 °C or 37 °C overnight with agitation. When needed, ampicillin was added to cultures. Cells were harvested and lysed as described above in the  6XHis-CsgD purification section. Protein concentration from lysates was determined by Bradford Assay. Roughly 0.04–0.05 mg of total proteins were loaded per well. SDS-PAGE was performed with 5% stacking and 12% resolving mini gels. Proteins were transferred on PVDF membranes for 1 h at 100 V in a Mini Trans-Blot Cell. A volume of 1 μg/mL of monoclonal mouse antibodies against CsgD were used to detect the protein of interest^32^. Goat antibodies against mouse IgG coupled with HRP were used as secondary antibodies at a dilution of 1:10,000 (GenScript). GroEL was detected using rabbit anti-GroEL at a dilution of 1:80,000 (GenScript) with rabbit anti-IgG coupled with HRP as secondary antibodies at 1:10,000 dilution (GenScript). Chemiluminescence was detected using SuperSignal West Pico PLUS (Thermo Fisher) and visualized with a Amersham Imager 600.

### Statistical analysis

All statistical analysis was performed using two-tailed unpaired parametric Student’s *t*-test and the mean ± SEM is represented using the software GraphPad Prism 8.0.1.

### Supplementary Information

Supplementary Information.

## Data Availability

The data generated during the current study are available from the corresponding author on reasonable request.
