# Supplementary material for: Differential regulatory control of curli (csg) gene expression in Salmonella enterica serovar Typhi requires more than a functional CsgD regulator
Source: Sci Rep. 2023 Sep 9;13:14905. doi: 10.1038/s41598-023-42027-y (PMC10492818; doi:10.1038/s41598-023-42027-y)
Supplement: Supplementary file 1 — Supplementary Information. [file 41598_2023_42027_MOESM1_ESM.pdf]

SUPPLEMENTARY MATERIALS:

Supplementary Table S1: List of bacterial strains and plasmids used in this study

Name	Strain	Characteristics	Source or reference
**Plasmid**			
	pRS415	Multicopy vector without promoter, *lacZ* reporter gene, ApR	Simons et al. (1987) [1]
p*csgB*$_{\mathrm{STy}}$-*lacZ*	DEF964	pRS415 with the promoter region of ISP1820 *csgB*	This study
p*csgB*$_{\mathrm{STm}}$-*lacZ*	pSIF570	pRS415 with the promoter region of SL1344 *csgB*	This study
p*csgD*$_{\mathrm{STy}}$-*lacZ*	pSIF584	pRS415 with the promoter region of ISP1820 *csgD*	This study
p*csgD*$_{\mathrm{STm}}$-*lacZ*	pSIF579	pRS415 with the promoter region of SL1344 *csgD*	This study
	pMEG375	Suicide vector, *sacRB, mobRP4, oriR6K*, CmR, ApR	R. Curtiss III
pMEG-Δ*csgD*	pSIF181	pMEG375 with flanking region of *csgD* for gene deletion	This study
pMEG-*csgD*$_{\mathrm{STy}}$	pSIF586	pMEG375 with ISP1820 *csgD* for gene permutation	This study
pMEG-*csgD*$_{\mathrm{STm}}$	pSIF585	pMEG375 with SL1344 *csgD* for gene permutation	This study
	pWSK29	Low copy vector, *lacZ, p*SC101 *ori*, ApR	Wang & Kushner (1991)[2]
pWSK-*csgD*$_{\mathrm{STy}}$	pSIF542	pWSK29 with ISP1820 *csgD*	This study
pWSK-*csgD*$_{\mathrm{STm}}$	pSIF541	pWSK29 with SL1344 *csgD*	This study
	pET14b	*ori*, T7 RNA polymerase, 6xHis-tag, ApR	Novagen
pET-*csgD*$_{\mathrm{STy}}$	pSIF619	pET14b carrying *csgD* of ISP1820	This study

pET-*csgD*$_{STm}$	pSIF618	pET14b carrying *csgD* of SL1344	This study
pWSK-*csg*$_{STm}$	pSIF641	pWSK29 with SL1344 complete curli operons (*csgBAC* and *csgDEFG*)	This study
Escherichia coli			
	DH5 alpha pir	endA1 hsdR17 glnV44 (= supE44) thi-1 recA1 gyrA96 relA1 φ80dlacΔ(lacZ)M15 Δ(lacZYA-argF)U169 zdg-232::Tn10 uidA::pir+	Invitrogen
	MGN-617 (X7213)	*thi-1, thr-1, leuB6, glnV44, fhuA21, lacY1, recA1, RP4-2-Tc::Mu λpir, ΔasdA4, Δzhf-2::Tn10*	Kaniga *et al.* (1998)[3]
	BL21 (DE3)	F– *ompT* *hsdS*$_B$ (r_B–, m_B–) *gal dcm* (DE3)	F W Studier et al. (1986)[4]
***Salmonella enterica* serovar Typhi**			
*S*Ty	DEF1045	ISP1820 Wild type	Hone *et al*. (1991)[5]
Δ*csgD*$_{Sty}$	DEF587	ISP1820 with *csgD* deletion	This study
*S*Tm pRS	DEF1153	*S*TY with pRS415	This study
*S*Ty p*csgB*$_{STy}$-*lacZ*	DEF1129	*S*TY with p*csgB*$_{STy}$-*lacZ* (DEF964)	This study
*S*Ty p*csgB*$_{STm}$-*lacZ*	DEF1557	*S*TY with p*csgB*$_{STm}$-*lacZ* (pSIF570)	This study
*S*Ty p*csgD*$_{STy}$-*lacZ*	DEF1685	*S*TY with p*csgD*$_{STy}$-*lacZ* (pSIF584)	This study
*S*Ty p*csgD*$_{STm}$-*lacZ*	DEF1669	*S*TY with p*csgB*$_{STm}$-*lacZ* (pSIF579)	This study
Δ*csgD*$_{STy}$ pWSK-	DEF1477	Δ*csgD*$_{STy}$ with pWSK-*csgD*$_{STy}$ (pSIF542)	This study

csgD$_{\mathrm{STy}}$			
Δ*csgD*$_{\mathrm{STy}}$ pWSK-*csgD*$_{\mathrm{STm}}$	DEF1478	Δ*csgD*$_{\mathrm{STy}}$ with pWSK-*csgD*$_{\mathrm{STm}}$ (pSIF541)	This study
CsgD$_{\mathrm{STm}}$	DEF1671	Genomic permutation of *S.* Typhi mutant Δ*csgD*$_{\mathrm{STy}}$ with *S.* Typhimurium *csgD*$_{\mathrm{STm}}$ performed with pMEG-*csgD*$_{\mathrm{STm}}$	This study
Δ*csgD*$_{\mathrm{STy}}$ CsgD$_{\mathrm{STy}}$	DEF1711	Genomic permutation of *S.* Typhi mutant Δ*csgD*$_{\mathrm{STy}}$ with *S.* Typhi*csgD*$_{\mathrm{STy}}$ performed with pMEG-*csgD*$_{\mathrm{STy}}$	This study
CsgD$_{\mathrm{STm}}$ p*csgB*$_{\mathrm{STm}}$-*lacZ*	DEF1734	CsgD$_{\mathrm{STm}}$ with p*csgB*$_{\mathrm{STm}}$-*lacZ* (pSIF570)	This study
Δ*csgD*$_{\mathrm{STy}}$ CsgD$_{\mathrm{STy}}$ p*csgB*$_{\mathrm{STy}}$-*lacZ*	DEF1731	*S*Ty CsgD$_{\mathrm{STy}}$ with p*csgB*$_{\mathrm{STy}}$-*lacZ* (DEF964)	This study
Δ*csg*$_{\mathrm{STy}}$ pWSK-*csg*$_{\mathrm{STm}}$	DEF1998	Δ*csgD*$_{\mathrm{STy}}$ with pWSK-*csg*$_{\mathrm{STm}}$ (pSIF641)	This study
Δ*tviB*	DEF442	ISP1820 with *tviB* deletion	This study
Δ*tviB* pWSK-*csg*$_{\mathrm{STm}}$	DEF1999	Δ*tviB* with pWSK-*csg*$_{\mathrm{STm}}$ (pSIF641)	This study
Ty2	DEF472	Ty2 Wild type	Weil & Felix (1920)[6]
SarB63	DEF304	SarB63 Wild type	Boyd et *al.* (1993)[7]
SarB64	DEF305	SarB64 Wild type	Boyd et *al.* (1993)[7]
Ty2 pWSK-*csg*$_{\mathrm{STm}}$	DEF2049	Ty2 with pWSK-*csg*$_{\mathrm{STm}}$ (pSIF641)	This study
SarB63	DEF2050	SarB63 with pWSK-*csg*$_{\mathrm{STm}}$ (pSIF641)	This study

pWSK-*csg*$_{STm}$			
SarB64 pWSK-*csg*$_{STm}$	DEF2051	SarB64 with pWSK-*csg*$_{STm}$ (pSIF641)	This study
***Salmonella enterica* serovar Typhimurium**			
*S*Tm	DEF1041	SL1344 Wild type	Hoiseth & Stocker (1981)[8]
Δ*csgD*$_{STm}$	DEF1501	SL1344 with *csgD* deletion	This study
*S*Tm pRS	DEF1549	*S*Tm with pRS415	This study
*S*Tm p*csgB*$_{STy}$-*lacZ*	DEF1474	*S*Tm with p*csgB*$_{STy}$-*lacZ* (DEF964)	This study
*S*Tm p*csgB*$_{STm}$-*lacZ*	DEF1558	*S*Tm with p*csgB*$_{STm}$-*lacZ* (pSIF570)	This study
*S*Tm p*csgD*$_{STy}$-*lacZ*	DEF1680	*S*Tm with p*csgD*$_{STy}$-*lacZ* (pSIF584)	This study
*S*Tm p*csgD*$_{STm}$-*lacZ*	DEF1679	*S*Tm with p*csgD*$_{STm}$-*lacZ* (pSIF579)	This study
Δ*csgD*$_{STm}$ pWSK-*csgD*$_{STy}$	DEF1551	Δ*csgD*$_{STm}$ with pWSK-*csgD*$_{STy}$ (pSIF542)	This study
Δ*csgD*$_{STm}$ pWSK-*csgD*$_{STm}$	DEF1552	Δ*csgD*$_{STm}$ with pWSK-*csgD*$_{STm}$ (pSIF541)	This study
CsgD$_{STy}$	DEF1675	Genomic permutation of *S*. Typhimurium mutant Δ*csgD*$_{STm}$ with *S*. Typhi *csgD*$_{STy}$ performed with pMEG-*csgD*$_{STy}$	This study
Δ*csgD*$_{STm}$	DEF1712	Genomic permutation of *S*. Typhimurium mutant	This study

CsgD$_{\mathrm{STm}}$		Δ*csgD*$_{\mathrm{STm}}$ with *S.* Typhimurium *csgD*$_{\mathrm{STm}}$ performed with pMEG-*csgD*$_{\mathrm{STm}}$	
CsgD$_{\mathrm{STy}}$ p*csgB*$_{\mathrm{STy}}$-*lacZ*	DEF1738	CsgD$_{\mathrm{STy}}$ with p*csgB*$_{\mathrm{STm}}$-*lacZ* (DEF964)	This study
Δ*csgD*$_{\mathrm{STm}}$ CsgD$_{\mathrm{STm}}$ p*csgB*$_{\mathrm{STm}}$-*lacZ*	DEF1737	*S*Tm CsgD$_{\mathrm{STm}}$ with p*csgB*$_{\mathrm{STm}}$-*lacZ* (pSIF570)	This study
Δ*csg*$_{\mathrm{STm}}$ pWSK-*csg*$_{\mathrm{STm}}$	DEF1997	Δ*csgD*$_{\mathrm{STm}}$ with pWSK-*csg*$_{\mathrm{STm}}$ (pSIF641)	This study

Supplementary Table S2: List of primers used in this study

Name	Sequence 5'-3'
csg_prom_F_short_EcoRI	gcgaattcAGATGTTGCACTGCTGTGGGTT
csg_prom_R_BamHI	gcggatccGCGCACCCAGTATTGTCAACAT
csgD_Prom_F_EcoRI	cagaattcGTGTATCGCGCACCTAAAA
csgD_Prom_R_BamHI	cgggatccTGCAGTTTTCCGGTTATGGC
csgD_F1	cgggatccCAGCTGTCAGATGTGCGATT
csgD_R2	actgcctggTGCAGTTTTCCGGTTATGGC
csgD_F3	aaaactgcaCCAGGCAGTTTCATGGGCAA
csgD_R4	aaggaaaaaagcggccgcCATGTCCTATCGAAGAGACG
csgD_swap F	cgggatccGCAATAACAGCGAAATGTACAAC
csgD_swap_R	gctctagaCGCGCTAAATCACTCGTACT
csgD_Prom_F_ BamHI	cgggatccGTGTATCGCGCACCTAAAA
csgD_R_XbaI	gctctagaCCAGGTCAGATAGCGTTTCA
CsgD-F-NdeI	ggaattccatATGTTTAATGAAGTCCATAGTAGTCA
CsgD-R-BamHI	cgggatccTTACCGCCTGAGATTATCGTTT
LacZ_alpha_R	GGCTGCGCAACTGTTGGG
curli-inter-F-FAM	/56-FAM/CGCACCCAGTATTGTCAACAT

curli-inter-R	CCCATGGGAAGCATAAGAAC
Csg_F	TGGGGCTAATCTTTGGCTAT
Csg_R	GCCTATGGCAGGGATATTTT

Supplementary Figure S1- Comparison between *S.* Typhi and *S.* Typhimurium of nucleotides and amino acids sequences of the assembly components of curli fimbriae

Comparison was performed using MUSCLE (Multiple Sequence Comparison by Log-Expectation) from European Bioinformatics Institute (ebi), comparing *S.* Typhi CT18 (STY) and *S.* Typhimurium SL1344 (STM) sequence taken from Pubmed Gene and Protein database. Homology between serovars are marked with an asterisk (*) and single-nucleotide polymorphisms are in green (missense), red (nonsense) and yellow (silent). The affected correspondent amino acids from missense and nonsense polymorphisms are also marked in green and red.

CsgG :

```
Nucleotide Sequence :

STY             ATGCCGCGCTTACTTATTTTGGTTGCCGTTTTATTGTTGAGCGGATGCTTAACTGCCCCG
STM             ATGCCGCGCTTACTTATTTTGGTTGCCGTTTTATTGTTGAGCGGATGCTTAACTGCCCCG
                ************************************************************

STY             CCGAAACAAGCTGCGAAACCGACATTAATGCCCCGCGCACAAAGTTACAAAGATTTGACG
STM             CCGAAACAAGCTGCGAAACCGACATTAATGCCCCGCGCACAAAGTTACAAAGATTTGACG
                ************************************************************

STY             CACTTACCTGCTCCCACCGGTAAGATCTTTGTTTCGGTATATAACATTCAGGATGAAACG
STM             CACTTACCTGCTCCCACCGGTAAGATCTTTGTTTCGGTATATAACATTCAGGATGAAACG
                ************************************************************

STY             GGCCAATTTAAACCTTACCCGGCAAGTAACTTTTCCACGGCTGTGCCGCAGAGCGCCACC
STM             GGCCAATTTAAACCTTACCCGGCAAGTAACTTTTCTACGGCTGTGCCGCAGAGCGCCACC
                *********************************** ************************

STY             GCTATGTTGGTCACCGCGCTGAAAGATTCGCGCTGGTTTATCCCACTAGAACGACAAGGC
STM             GCTATGTTGGTCACTGCGCTGAAAGATTCGCGCTGGTTTATCCCGCTGGAACGACAAGGC
                ************** ***************************** ** ************
```

```
STY    TTACAGAATCTTTTGAATGAACGGAAAATTATTCGCGCAGCCCAGGAAAACGGCACCGTG
STM    TTACAGAATCTTTTGAATGAACGGAAAATTATTCGCGCAGCCCAGGAAAACGGCACCGTG
       ************************************************************

STY    GCGATGAATAACCGTATCCCGCTTCAGTCGTTGACGGCGGCAAATATTATGGTGGAAGGT
STM    GCGATGAATAACCGTATCCCGCTTCAGTCGCTGACGGCGGCAAATATTATGGTGGAAGGT
       ****************************** *****************************

STY    TCTATTATTGGTTATGAAAGTAACGTCAAATCCGGCGGGGTCGGCGCAAGATATTTCGGT
STM    TCTATTATTGGTTATGAAAGTAACGTCAAATCCGGCGGGGTCGGCGCAAGATATTTCGGT
       ************************************************************

STY    ATTGGCGCCGATACGCAGTATCAGCTGGATCAGATTGCTGTCAACCTGCGCGTGGTTAAC
STM    ATTGGCGCCGATACGCAGTATCAGCTGGATCAGATTGCTGTCAACCTGCGCGTGGTTAAC
       ************************************************************

STY    GTCAGTACGGGCGAGATCCTTTCTTCGGTGAACACCAGTAAAACGATCCTTTCCTATGAA
STM    GTCAGTACGGGCGAGATCCTTTCCTCGGTGAACACCAGTAAAACGATCCTTTCCTATGAA
       *********************** ************************************

STY    GTACAGGCAGGCGTGTTCCGTTTTATTGATTACCAGCGCTTACTGGAAGGCGAAATCGGC
STM    GTACAGGCAGGCGTGTTCCGTTTTATTGATTACCAGCGCTTACTGGAAGGCGAAATCGGC
       ************************************************************

STY    TATACCTCGAACGAACCGGTGATGCTGTGTCTGATGTCAGCCATTGAAACCGGCGTTATC
STM    TATACCTCGAACGAACCGGTGATGCTGTGTCTGATGTCAGCCATTGAAACCGGCGTTATC
       ************************************************************

STY    TTCCTCATTAATGATGGTATCGATCGCGGACTGTGGGATTTGCAGAATAAAGCGGACAGG
STM    TTCCTCATTAATGATGGTATCGATCGCGGACTGTGGGATTTGCAGAATAAAGCGGACAGG
       ************************************************************

STY    CAAAATGATATTCTGGTGAAATACCGTGAGCTGTCAGTACCGCCAGAATCCTGA
STM    CAAAATGATATTCTGGTGAAATACCGTGAGCTGTCAGTACCGCCAGAATCCTGA
       ******************************************************
```

Amino Acid Sequence :

```
STY    MPRLLILVAVLLLSGCLTAPPKQAAKPTLMPRAQSYKDLTHLPAPTGKIFVSVYNIQDET
STM    MPRLLILVAVLLLSGCLTAPPKQAAKPTLMPRAQSYKDLTHLPAPTGKIFVSVYNIQDET
       ************************************************************
```

```
STY             GQFKPYPASNFSTAVPQSATAMLVTALKDSRWFIPLERQGLQNLLNERKIIRAAQENGTV
STM             GQFKPYPASNFSTAVPQSATAMLVTALKDSRWFIPLERQGLQNLLNERKIIRAAQENGTV
                ************************************************************

STY             AMNNRIPLQSLTAANIMVEGSIIGYESNVKSGGVGARYFGIGADTQYQLDQIAVNLRVVN
STM             AMNNRIPLQSLTAANIMVEGSIIGYESNVKSGGVGARYFGIGADTQYQLDQIAVNLRVVN
                ************************************************************

STY             VSTGEILSSVNTSKTILSYEVQAGVFRFIDYQRLLEGEIGYTSNEPVMLCLMSAIETGVI
STM             VSTGEILSSVNTSKTILSYEVQAGVFRFIDYQRLLEGEIGYTSNEPVMLCLMSAIETGVI
                ************************************************************

STY             FLINDGIDRGLWDLQNKADRQNDILVKYRELSVPPES
STM             FLINDGIDRGLWDLQNKADRQNDILVKYRELSVPPES
                *************************************
```

CsgF :

Nucleotide Sequence :

```
STY             ATGCGTGTTAAACATGCAGTAGTGCTGCTCATGCTTTTTTCGCCATTAACCTGGGCTGGA
STM             ATGCGTGTTAAACATGCAGTAGTGCTGCTCATGCTTTTTTCGCCATTAACCTGGGCTGGA
                ************************************************************

STY             AATATGACGTTCCAGTTCCGTAATCCTAACTTTGGTGGAAACCCCAATAACGGTTCCTTT
STM             AATATGACGTTCCAGTTCCGTAATCCTAACTTTGGTGGAAACCCCAATAACGGTTCCTTT
                ************************************************************

STY             TTATTGAATAGCGCCCAGGCGCAAAATTCATATAAAGACCCCGCTTATGATAACGATTTT
STM             TTATTGAATAGCGCCCAGGCGCAAAATTCATATAAAGACCCCGCTTATGATAACGATTTT
                ************************************************************

STY             GGTATCGAGACCCCCTCAGCGTTGGATAACTTTACGCAGGCTATTCAATCGCAAATTCTG
STM             GGTATCGAAACCCCCTCAGCGTTGGATAACTTTACGCAGGCTATTCAATCGCAAATTCTG
                ******** ***************************************************

STY             GGCGGCTTGTTGACCAATATTAATACCGGAAAACCAGGACGTATGGTGACCAATGATTTT
STM             GGCGGCTTGTTGACCAATATTAATACCGGGAAACCAGGACGTATGGTGACCAACGATTTT
                ***************************** *********************** ******

STY             ATTATCGATATCGCTAATCGCGACGGACAGCTCCAGCTCAACGTCACGGACAGAAAAACG
```

STM ATTATCGATATCGCTAATCGCGACGGACAGCTCCAGCTCAACGTCACGGACAGAAAAACG
****

STY GGAAGAACCTCGACCATCGAAGTGTCAGGTTTACAAACTCAGTCAACCGATTTTTAA
STM GGAAGAACCTCGACCATCGAAGTGTCAGGTTTACAAACTCAGTCAACCGATTTTTAA
****

Amino Acid Sequence :

STY MRVKHAVVLLMLFSPLTWAGNMTFQFRNPNFGGNPNNGSFLLNSAQAQNSYKDPAYDNDF
STM MRVKHAVVLLMLFSPLTWAGNMTFQFRNPNFGGNPNNGSFLLNSAQAQNSYKDPAYDNDF
****

STY GIETPSALDNFTQAIQSQILGGLLTNINTGKPGRMVTNDFIIDIANRDGQLQLNVTDRKT
STM GIETPSALDNFTQAIQSQILGGLLTNINTGKPGRMVTNDFIIDIANRDGQLQLNVTDRKT
****

STY GRTSTIEVSGLQTQSTDF
STM GRTSTIEVSGLQTQSTDF
****

CsgE :

Nucleotide Sequence :

STY ATGAAACGCTATCTGACCTGGATTGTAGCAGCAGAGTTACTGTTCGCTACCGGAAACCTG
STM ATGAAACGCTATCTGACCTGGATTGTAGCAGCAGAGTTACTGTTCGCTACCGGAAACCTG
****

STY CATGCCAATGAAGTTGAAGTCGAGGTTCCCGGATTGTTAACCGACCATACCGTCTCTTCG
STM CATGCCAATGAAGTTGAAGTCGAGGTTCCCGGATTGTTAACCGACCATACCGTCTCTTCG
****

STY ATAGGACATGAATTCTATCGTGCATTCAGCGACAAATGGGAAAGCGAATACACCGGCAAT
STM ATAGGACATGAATTCTATCGTGCATTCAGCGACAAATGGGAAAGCGAATACACCGGCAAT
****

STY CTGACCATTAATGAAAGACCCAGTGCGCGTTGGGGAAGCTGGATCACCATAACGGTAAAT
STM CTGACCATTAATGAAAGACCCAGTGCGCGTTGGGGAAGCTGGATCACCATAACGGTAAAT
****

STY CAGGACGTTATTTTCCAGACCTTTTTATTTCCAATGAAAAGAGACTTCGAGAAAACCGTC
STM CAGGACGTTATTTTCCAGACCTTTTTATTTCCAATGAAAAGAGACTTCGAGAAAACCGTC

```
                ************************************************************

STY             GTCTTCGCATTAGCGCAAACAGAGGAAGCATTAAATCGCCGACAAATAGATCAAACGCTA
STM             GTCTTCGCATTAGCGCAAACAGAGGAAGCATTAAATCGCCGACAAATAGATCAAACGCTA
                ************************************************************

STY             TTAAGTACGAGTGATTTAGCGCGTGATGAATTCTAA
STM             TTAAGTACGAGTGATTTAGCGCGTGATGAATTCTAA
                ************************************
```

Amino Acid Sequence :

```
STY             MKRYLTWIVAAELLFATGNLHANEVEVEVPGLLTDHTVSSIGHEFYRAFSDKWESEYTGN
STM             MKRYLTWIVAAELLFATGNLHANEVEVEVPGLLTDHTVSSIGHEFYRAFSDKWESEYTGN
                ************************************************************

STY             LTINERPSARWGSWITITVNQDVIFQTFLFPMKRDFEKTVVFALAQTEEALNRRQIDQTL
STM             LTINERPSARWGSWITITVNQDVIFQTFLFPMKRDFEKTVVFALAQTEEALNRRQIDQTL
                ************************************************************

STY             LSTSDLARDEF
STM             LSTSDLARDEF
                ***********
```

CsgD :

Nucleotide Sequence :

```
STY             ATGTTTAATGAAGTCCATAGTAGTCATGGTCACACACTATTGTTGATCACAAAGCCATCT
STM             ATGTTTAATGAAGTCCATAGTAGTCATGGTCACACACTATTGTTGATCACAAAGCCATCT
                ************************************************************

STY             CTGCAAGCTACGGCATTATTGCAACATTTAAAGCAATCGCTGGCCATAACCGGAAAACTG
STM             CTGCAAGCTACGGCATTATTGCAACATTTAAAGCAATCGCTGGCCATAACCGGAAAACTG
                ************************************************************

STY             CATAATATTCAACGTTCTCTGGAAGATATCTCAGCCGGTTGCATTGTTTTAATGGATATG
STM             CATAATATTCAACGTTCTCTGGAAGATATCTCGGCCGGTTGCATTGTTTTAATGGATATG
                ******************************** ***************************

STY             ATGGAAGCGGATAAGAAGCTTATCCACTATTGGCAGGATAATTTAAGCCGCAAAAACAAT
STM             ATGGAAGCGGATAAGAAGCTTATCCACTATTGGCAGGATAATTTAAGCCGCAAAAACAAT
```

```
                    ************************************************************

STY                 AATATAAAAACATTATTGTTAAATACCCCTGACGATTATCCCTACCGTGAAATTGAAAAC
STM                 AATATAAAAACATTATTGTTAAATACCCCTGACGATTATCCCTACCGTGAAATTGAAAAC
                    ************************************************************

STY                 TGGCCTCATATTAACGGCGTGTTTTACGCCACTGAAGACCAGGAACACGTGGTCAGCGGA
STM                 TGGCCTCATATTAACGGCGTGTTTTACGCTACTGAAGACCAGGAACACGTGGTCAGCGGA
                    ***************************** ******************************

STY                 TTACAGGGTATTCTGCGGGGCGAATGCTATTTTTCACAAAAATTAGCCAGTTACCTGATT
STM                 TTACAGGGTATTCTGCGTGGCGAATGCTATTTTTCACAAAAATTAGCCAGTTACCTGATT
                    ***************** ******************************************

STY                 ACACACTCAGGAAATTACCGCTACAACAGCACCGAGTCCGCATTACTCACTCATCGCGAA
STM                 ACGCACTCAGGAAATTACCGCTACAACAGCACCGAGTCCGCATTACTCACTCATCGCGAA
                    ** *********************************************************

STY                 AAAGAGATCCTCAATAAGTTACGTATTGGTGCCTCTAATAATGAAATCGCCAGGTCGCTA
STM                 AAAGAGATCCTCAATAAGTTACGTATTGGTGCCTCTAATAATGAAATCGCGAGGTCGCTA
                    ************************************************** *********

STY                 TTTATCAGCGAGAATACGGTTAAGACACATCTTTATAATCTTTTCAAAAAGATAGCTGTC
STM                 TTTATCAGCGAGAATACGGTTAAGACACATCTTTATAATCTTTTCAAAAAGATAGCTGTC
                    ************************************************************

STY                 AAAAATCGCACCCAGGCAGTTTCATGA------------------------
STM                 AAAAATCGCACCCAGGCAGTTTCATGGGCAAACGATAATCTCAGGCGGTAA
                    **************************
```

Amino Acid Sequence :

```
STY                 MFNEVHSSHGHTLLLITKPSLQATALLQHLKQSLAITGKLHNIQRSLEDISAGCIVLMDM
STM                 MFNEVHSSHGHTLLLITKPSLQATALLQHLKQSLAITGKLHNIQRSLEDISAGCIVLMDM
                    ************************************************************

STY                 MEADKKLIHYWQDNLSRKNNNIKTLLLNTPDDYPYREIENWPHINGVFYATEDQEHVVSG
STM                 MEADKKLIHYWQDNLSRKNNNIKTLLLNTPDDYPYREIENWPHINGVFYATEDQEHVVSG
                    ************************************************************

STY                 LQGILRGECYFSQKLASYLITHSGNYRYNSTESALLTHREKEILNKLRIGASNNEIARSL
STM                 LQGILRGECYFSQKLASYLITHSGNYRYNSTESALLTHREKEILNKLRIGASNNEIARSL
```

```
                ************************************************************

STY             FISENTVKTHLYNLFKKIAVKNRTQAVS--------
STM             FISENTVKTHLYNLFKKIAVKNRTQAVSWANDNLRR
                ****************************
```

CsgB :

Nucleotide Sequence :

```
STY             ATGAAAAACAAATTGTTATTTATGATGTTGACAATACTGGGTGCGCCTGGGATTGCAACC
STM             ATGAAAAACAAATTGTTATTTATGATGTTGACAATACTGGGTGCGCCTGGGATTGCAACC
                ************************************************************

STY             GCGACAAATTATGATCTGGCTCGTTCAGAGTATAATTTTGCGGTAAATGAATTAAGCAAG
STM             GCGACAAATTATGATCTGGCTCGTTCAGAGTATAATTTTGCGGTAAATGAATTAAGCAAG
                ************************************************************

STY             TCTTCATTTAATCAGGCGGCCATTATTGGTCAAGTCGGCACGGATAATAGTGCCAGAGTA
STM             TCTTCATTTAATCAGGCGGCCATTATTGGTCAAGTCGGCACGGATAATAGTGCCAGAGTA
                ************************************************************

STY             CGCCAGGAAGGATCAAAACTATTGTCCGTTATTTCACAAGAAGGAGAAAATAATCGGGCG
STM             CGCCAGGAAGGATCAAAACTATTGTCCGTTATTTCACAAGAAGGAGGAAATAATCGGGCG
                ********************************************** *************

STY             AAAGTCGACCAGGCAGGGAATTATAACTTTGCGTATATTGAGCAAACGGGCAATGCCAAC
STM             AAAGTCGACCAGGCAGGGAATTATAACTTTGCGTATATTGAGCAAACGGGCAATGCCAAC
                ************************************************************

STY             GATGCCAGTATATCGCAAAGCGCTTACGGTAATAGTGCGGCTATTATCCAGAAAGGTTCT
STM             GATGCCAGTATATCGCAAAGCGCTTACGGTAATAGTGCGGCTATTATCCAGAAAGGTTCT
                ************************************************************

STY             GGAAATAAGGCCAATATTACCCAGTACGGTACGCAGAAAACAGCAGTTGTAGTGCAGAAA
STM             GGAAATAAGGCCAATATTACCCAGTACGGTACGCAGAAAACAGCAGTTGTAGTGCAGAAA
                ************************************************************

STY             CAGTCGCATATGGCTATTCGCGTCACCCAACGCTAA
STM             CAGTCGCATATGGCTATTCGCGTCACCCAACGCTAA
                ************************************
```

```
Amino Acid Sequence :

STY             MKNKLLFMMLTILGAPGIATATNYDLARSEYNFAVNELSKSSFNQAAIIGQVGTDNSARV
STM             MKNKLLFMMLTILGAPGIATATNYDLARSEYNFAVNELSKSSFNQAAIIGQVGTDNSARV
                ************************************************************

STY             RQEGSKLLSVISQEGENNRAKVDQAGNYNFAYIEQTGNANDASISQSAYGNSAAIIQKGS
STM             RQEGSKLLSVISQEGGNNRAKVDQAGNYNFAYIEQTGNANDASISQSAYGNSAAIIQKGS
                *************** ********************************************

STY             GNKANITQYGTQKTAVVVQKQSHMAIRVTQR
STM             GNKANITQYGTQKTAVVVQKQSHMAIRVTQR
                *******************************
```

CsgA :

```
Nucleotide Sequence :

STY             ATGAAACTTTTAAAAGTGGCAGCATTCGCAGCAATCGTAGTTTCTGGCAGTGCTCTGGCT
STM             ATGAAACTTTTAAAAGTGGCAGCATTCGCAGCAATCGTAGTTTCTGGCAGTGCTCTGGCT
                ************************************************************

STY             GGCGTCGTTCCACAATGGGGCGGCGGCGGTAATCATAACGGCGGCGGCAATAGTTCCGGG
STM             GGCGTCGTTCCACAATGGGGCGGCGGCGGTAATCATAACGGCGGCGGCAATAGTTCCGGC
                ***********************************************************

STY             CCGGATTCCACGTTGAGCATTTATCAGTACGGTTCCGCTAACGCTGCGCTTGCTCTGCAA
STM             CCGGATTCCACGTTGAGCATTTATCAGTACGGTTCCGCTAACGCTGCGCTTGCTCTGCAA
                ************************************************************

STY             AGCGATGCCCGTAAATCTGAAACGACCATTACCCAGAGCGGTTATGGTAACGGCGCCGAT
STM             AGCGATGCCCGTAAATCTGAAACGACCATTACCCAGAGCGGTTATGGTAACGGCGCCGAT
                ************************************************************

STY             GTAGGCCAGGGTGCGGATAACAGTACTATTGAACTGACTCAGAATGGTTTCAGAAACAAT
STM             GTAGGCCAGGGTGCGGATAACAGTACTATTGAACTGACTCAGAATGGTTTCAGAAACAAT
                ************************************************************

STY             GCCACCATCGACCAGTGGAACGCTAAAAACTCCGATATTACTGTCGGTCAATACGGCGGT
STM             GCCACCATCGACCAGTGGAACGCTAAAAACTCCGATATTACTGTCGGTCAATACGGCGGT
                ************************************************************
```

STY AATAACGCCGCGCTGGTTAATCAGACCGCATCTGATTCCAGCGTAATGGTGCGTCAGGTT

STM AATAACGCCGCGCTGGTTAATCAGACCGCATCTGATTCCAGCGTAATGGTGCGTCAGGTT

****

STY GGTTTTGGCAACAACGCCACGGCTAACCAGTATTAA

STM GGTTTTGGCAACAACGCCACGGCTAACCAGTATTAA

****

Amino Acid Sequence :

STY MKLLKVAAFAAIVVSGSALAGVVPQWGGGGNHNGGGNSSGPDSTLSIYQYGSANAALALQ

STM MKLLKVAAFAAIVVSGSALAGVVPQWGGGGNHNGGGNSSGPDSTLSIYQYGSANAALALQ

****

STY SDARKSETTITQSGYGNGADVGQGADNSTIELTQNGFRNNATIDQWNAKNSDITVGQYGG

STM SDARKSETTITQSGYGNGADVGQGADNSTIELTQNGFRNNATIDQWNAKNSDITVGQYGG

****

STY NNAALVNQTASDSSVMVRQVGFGNNATANQY

STM NNAALVNQTASDSSVMVRQVGFGNNATANQY

****

CsgC :

Nucleotide Sequence :

STY ATGCATACTTTATTGCTCCTTGCCGCACTTTCAAATCAGATTACGTTTACCACGACTCAG

STM ATGCATACTTTATTGCTCCTTGCCGCACTTTCAAATCAGATTACGTTTACCACGACTCAG

****

STY CAAGGCGATATTTACACGGTGATCCCTCAGGTCACATTAAACGAACCCTGCGTCTGTCTG

STM CAAGGCGATATTTACACGGTGATCCCTCAGGTCACATTAAACGAACCCTGCGTCTGTCAG

** *

STY GTGCAAATTCTCTCTGTGCGCGACGGCGTCGGGGGACAAAGCCATACACAGCAAAAACAA

STM GTGCAAATTCTCTCTGTGCGCGACGGCGTCGGGGGACAAAGCCATACACAGCAAAAACAA

****

STY ACGCTATCTTTACCTGCTAATCAACCGATTGAGTTGTCTCGTCTTAGTGTAAATATATCT

STM ACGCTATCTTTACCTGCTAATCAACCGATTGAGTTGTCTCGTCTTAGTGTAAATATATCT

****

STY TCAGAGGACTCGGTTAAAATTATTGTTACTGTTTCGGACGGACAATCACTGCATTTATCA

```
STM             TCAGAGGACTCGGTTAAAATTATTGTTACTGTTTCGGACGGACAATCACTGCATTTATCA
                ************************************************************

STY             CAACAATGGCCGCCTTCTGCACAGTAG
STM             CAACAATGGCCGCCTTCTGCACAGTAG
                ***************************

Amino Acid Sequence :

STY             MHTLLLLAALSNQITFTTTQQGDIYTVIPQVTLNEPCVCLVQILSVRDGVGGQSHTQQKQ
STM             MHTLLLLAALSNQITFTTTQQGDIYTVIPQVTLNEPCVCQVQILSVRDGVGGQSHTQQKQ
                *************************************** ********************

STY             TLSLPANQPIELSRLSVNISSEDSVKIIVTVSDGQSLHLSQQWPPSAQ
STM             TLSLPANQPIELSRLSVNISSEDSVKIIVTVSDGQSLHLSQQWPPSAQ
                ************************************************
```

Supplementary Figure S2- Monoclonal antibodies anti-*csgD* binding to 6His-CsgD. Western Blot performed using monoclonal anti-*csgD* against 6XHis-CsgD$_{STy}$ and 6XHis-CsgD$_{STm}$.

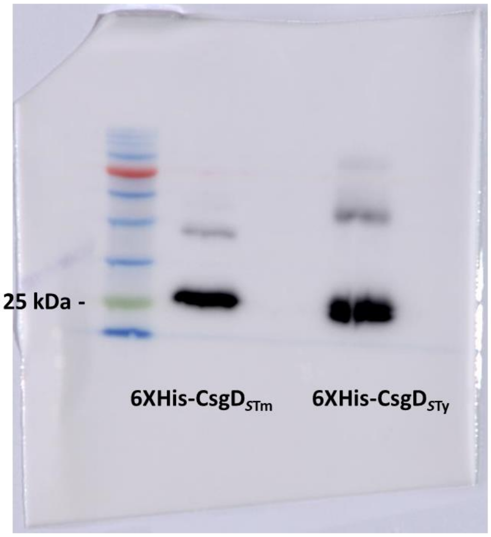

Supplementary Figure S3- Original full length gels and Western Blot. a) EMSA gels, related to Figure 4. b-c) Western Blot membranes, related to Figure 6. d) Western Blot membranes, related to Figure 7.

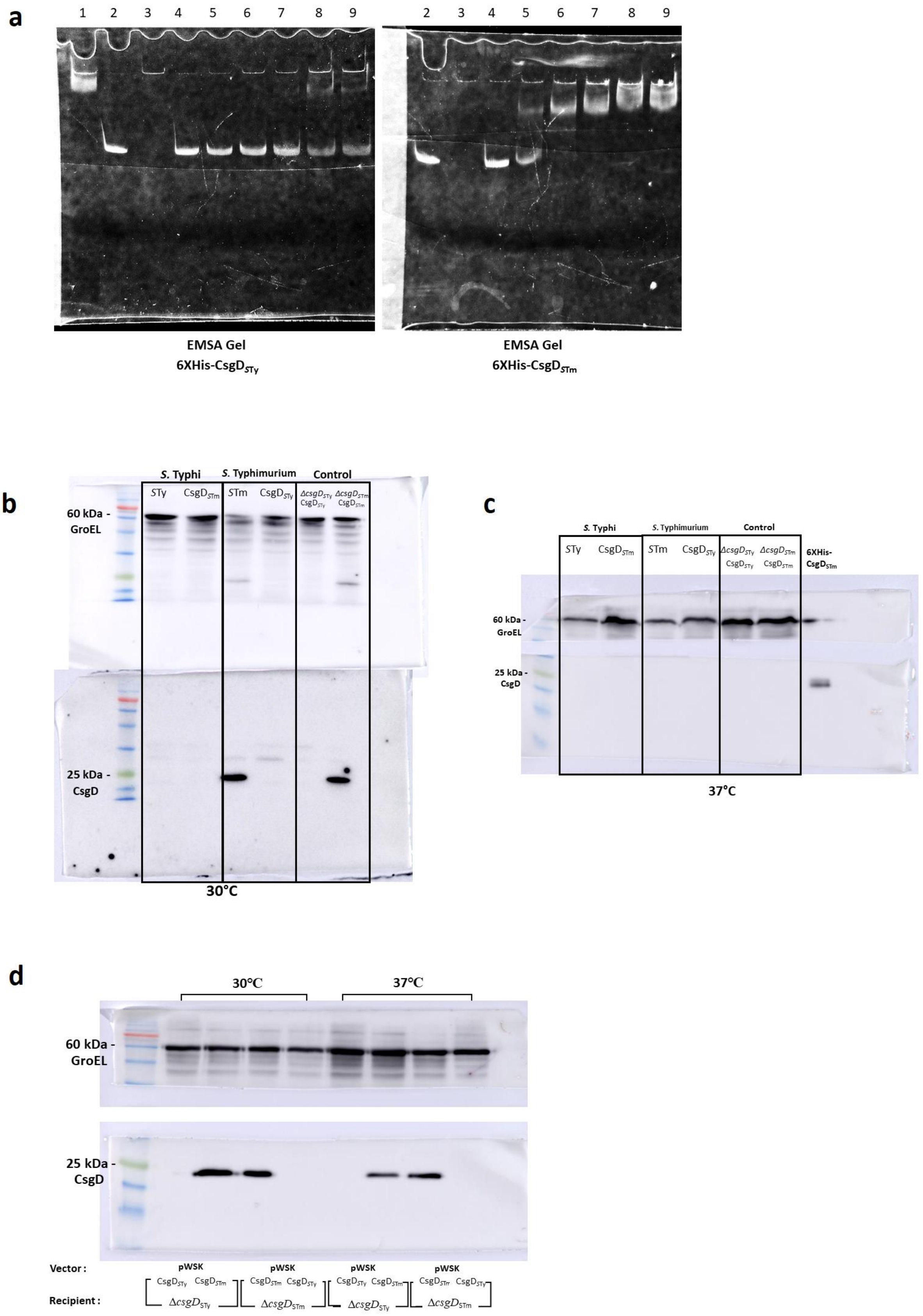
a
1 2 3 4 5 6 7 8 9
2 3 4 5 6 7 8 9
EMSA Gel
6XHis-CsgD$_{STy}$
EMSA Gel
6XHis-CsgD$_{STm}$
b
S. Typhi
S. Typhimurium
Control
*S*Ty
CsgD$_{STm}$
*S*Tm
CsgD$_{STy}$
ΔcsgD$_{STy}$ CsgD$_{STy}$
ΔcsgD$_{STm}$ CsgD$_{STm}$
60 kDa - GroEL
25 kDa - CsgD
30°C
c
S. Typhi
S. Typhimurium
Control
*S*Ty
CsgD$_{STm}$
*S*Tm
CsgD$_{STy}$
ΔcsgD$_{STy}$ CsgD$_{STy}$
ΔcsgD$_{STm}$ CsgD$_{STm}$
6XHis-CsgD$_{STm}$
60 kDa - GroEL
25 kDa - CsgD
37°C
d
30°C
37°C
60 kDa - GroEL
25 kDa - CsgD
Vector :
pWSK
CsgD$_{STy}$ CsgD$_{STm}$
pWSK
CsgD$_{STm}$ CsgD$_{STy}$
pWSK
CsgD$_{STy}$ CsgD$_{STm}$
pWSK
CsgD$_{STm}$ CsgD$_{STy}$
Recipient :
ΔcsgD$_{STy}$
ΔcsgD$_{STm}$
ΔcsgD$_{STy}$
ΔcsgD$_{STm}$

REFERENCE:

1 Simons, R. W., Houman, F. & Kleckner, N. Improved single and multicopy *lac*-based cloning vectors for protein and operon fusions. *Gene* **53**, 85-96, doi:10.1016/0378-1119(87)90095-3 (1987).

2 Wang, R. F. & Kushner, S. R. Construction of versatile low-copy-number vectors for cloning, sequencing and gene expression in *Escherichia coli*. *Gene* **100**, 195-199 (1991).

3 Kaniga, K., Compton, M. S., Curtiss, R. 3rd & Sundaram, P. Molecular and functional characterization of *Salmonella enterica* serovar typhimurium *poxA* gene: effect on attenuation of virulence and protection. *Infection and immunity* **66**, 5599-5606, doi:10.1128/iai.66.12.5599-5606.1998 (1998).

4 Studier, F. W. & Moffatt, B. A. Use of bacteriophage T7 RNA polymerase to direct selective high-level expression of cloned genes. *Journal of molecular biology* **189**, 113-130, doi:10.1016/0022-2836(86)90385-2 (1986).

5 Hone, D. M., Harris, A. M., Chatfield, S., Dougan, G. & Levine, M. M. Construction of genetically defined double aro mutants of *Salmonella typhi*. *Vaccine* **9**, 810-816, doi:10.1016/0264-410x(91)90218-u (1991).

6 Weil, E. & Felix, A. Ueber den doppeltypus der rezeptoren in der typhus-paratyphus-gruppe. *Zeitschr. Immunitaetsforsch* **29**, 24 (1920).

7 Boyd, E. F. *et al. Salmonella* reference collection B (SARB): strains of 37 serovars of subspecies I. *Microbiology (Reading, England)* **139**, 1125-1132, doi:https://doi.org/10.1099/00221287-139-6-1125 (1993).

8 Hoiseth, S. K. & Stocker, B. A. Aromatic-dependent Salmonella typhimurium are non-virulent and effective as live vaccines. *Nature* **291**, 238-239, doi:10.1038/291238a0 (1981).
